# Prognostic and immunological roles of IL18RAP in human cancers

**DOI:** 10.18632/aging.205017

**Published:** 2023-09-11

**Authors:** Wu Chen, Tianbao Song, Fan Zou, Yuqi Xia, Ji Xing, Weimin Yu, Ting Rao, Xiangjun Zhou, Chenglong Li, Jinzhuo Ning, Sheng Zhao, Yuan Ruan, Fan Cheng

**Affiliations:** 1 Department of Urology, Hubei International Scientific and Technological Cooperation Base of Immunotherapy, Renmin Hospital of Wuhan University, Wuhan 430000, Hubei, P.R. China

**Keywords:** IL18 receptor accessory protein, pan-cancer analysis, bioinformatics, immune infiltration, immunotherapy

## Abstract

Across several cancers, IL18 receptor accessory protein (IL18RAP) is abnormally expressed, and this abnormality is related to tumor immunity and heterogeneous clinical outcomes. In this study, based on bioinformatics analysis, we discovered that IL18RAP is related to the human tumor microenvironment and promotes various immune cells infiltration. Additionally, the multiple immunofluorescence staining revealed that with the increased expression of IL18RAP, the number of infiltrated M1 macrophages increased. This finding was confirmed by coculture migration analysis using three human cancer cell lines (MDA-MB-231, U251, and HepG2) with IL18RAP knockdown. We discovered a positive link between IL18RAP and the majority of immunostimulators, immunoinhibitors, major histocompatibility complex (MHC) molecules, chemokines, and chemokine receptor genes using Spearman correlation analysis. Additionally, functional IL18RAP’s gene set enrichment analysis (GSEA) revealed that it is related to a variety of immunological processes, such as positive regulation of interferon gamma production and positive regulation of NK cell-mediated immunity. Moreover, we used single-cell RNA sequencing analysis to detect that IL18RAP was mainly expressed in immune cells, and HALLMARK analysis confirmed that the INF-γ gene set expression was upregulated in CD8Tex cells. In addition, in human and mouse cancer cohorts, we found that the level of IL18RAP can predict the immunotherapy response. In short, our study showed that IL18RAP is a new tumor biomarker and may become a potential immunotherapeutic target in cancer.

## INTRODUCTION

Today, cancer is a severe threat to social health and a leading cause of death [1]. Globally, there were over 19.3 million new cancer diagnoses and around 10 million cancer-related deaths in 2020, according to the Global Cancer Statistics report [2].

Despite significant advancements in therapy, patients still do not feel pleased with cancer care due to ineffective treatment outcomes, substantial drug side effects, drug resistance, and high treatment costs. In the past decade, immunotherapy treatment for cancer has made great breakthroughs, among which the progress of immune checkpoint inhibitors (ICIs) is the most remarkable. Since anti-cytotoxic T-lymphocyte-associated protein 4 (anti-CTLA4) was approved for advanced melanoma treatment in 2011, ICIs have rapidly gained approval and been used to treat various cancers, which has led to an unprecedented increase in survival [3–5]. However, the efficacy of most immunotherapies is still limited by specific tumor types and specific genetic mutations. Therefore, finding new treatment targets and possible tumor biomarkers is crucial. It is now easier to examine the association between individual genes and cancer survival, prognosis, and immune infiltration thanks to the growth of numerous cancer datasets like Genotype-Tissue Expression (GTEx) and The Cancer Genome Atlas (TCGA).

IL18, an IL1-related cytokine that is crucial for both adaptive and innate immunity, is widely known [6]. Initial studies confirmed that IL18 is secreted by macrophages and stimulates IFN-γ production by synergistic interaction with IL12 [7, 8]. In addition, IL18 enhances natural killer (NK) cell lethality, as well as Th1, Th2, and Th17 responses [9–12]. However, the action of IL18 requires binding to the specific receptor IL18R1. Although it does not directly mediate IL18 binding, IL18 receptor accessory protein (IL18RAP) is critical for IL18 signaling [13]. It has been shown that in the absence of IL18RAP, IL-18 is unable to stimulate Th1 cells to produce IFN-γ. In addition, it has been shown that neutrophils lacking IL18RAP do not respond to IL18, which affects neutrophil activation and cytokines production [14]. Due to its special function, IL18RAP has attracted more attention these years. Given the significance of IL18RAP in immunological regulation, numerous researchers have examined how it affects diseases such as asthma, rheumatoid arthritis, and Crohn’s disease [15–17]. It is well recognized that the tumor microenvironment (TME) is made up of a variety of complex elements, among which are immune cells. As a result of IL18RAP’s ability to modulate the immune system, its function in malignancies has also drawn attention. For example, Zhu et al. revealed that the IL18RAP polymorphism may cause esophageal cancer [18]. Wang et al. also confirmed that IL18RAP, as a key prognostic gene, was highly associated with the prognosis of hepatocellular carcinoma [19]. However, the expression of IL18RAP across cancers are still unclear, and its clinical significance and molecular biological role remain to be investigated.

Using the GTEx and TCGA databases, we investigated the IL18RAP expression in 33 different human cancers. Additionally, this study emphasized the connection between pancancer-level IL18RAP expression and clinical prognosis, DNA methylation, tumor mutation burden (TMB), TME, microsatellite instability (MSI), and immunotherapy.

## RESULTS

### IL18RAP mRNA expression levels in various normal and cancer tissues

**Figure 1:**
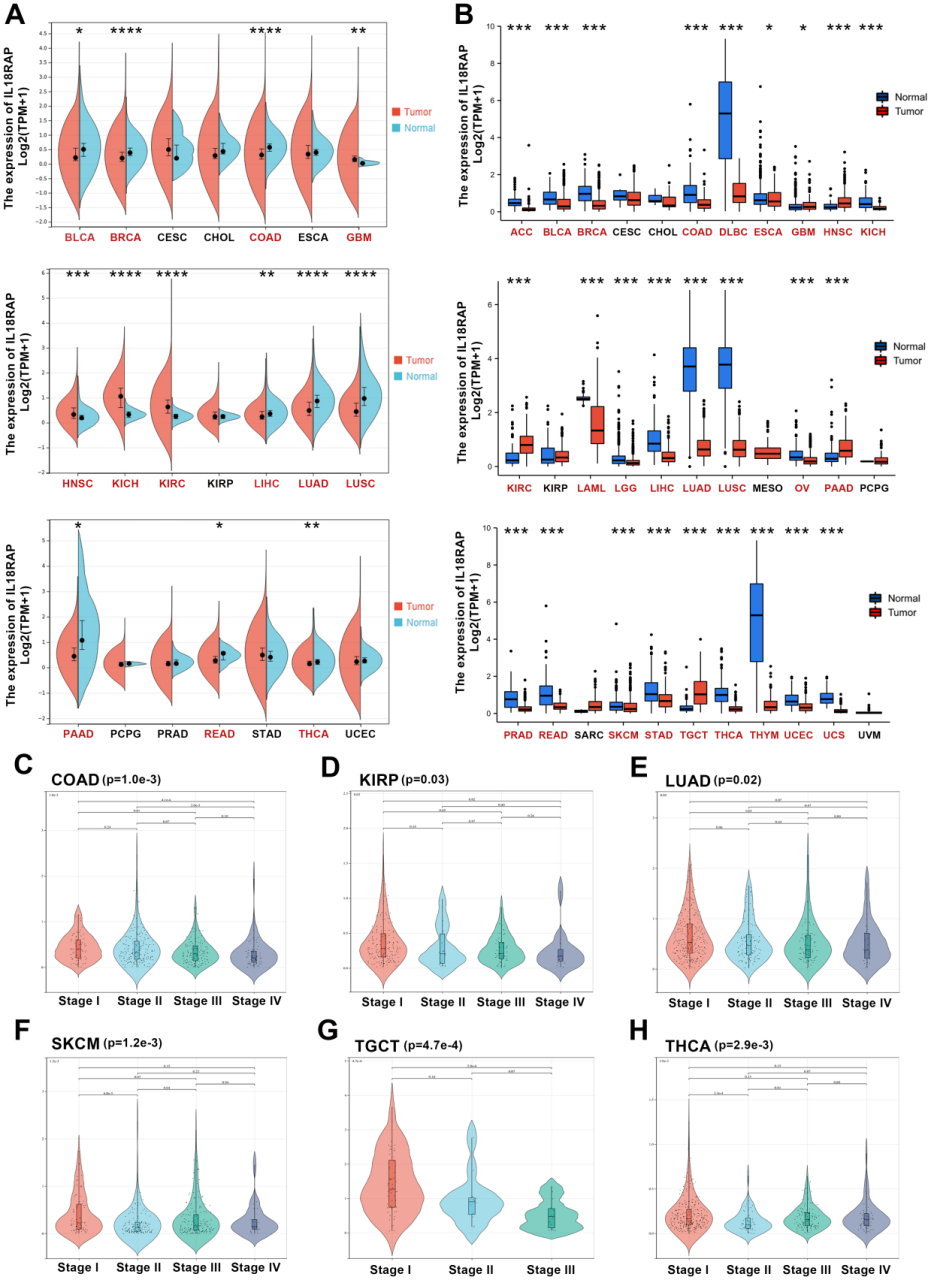
**Differential expression analysis of IL18RAP.** (**A**) The mRNA expression of IL18RAP in pan-cancer. (**B**) The mRNA expression of IL18RAP was examined across cancers and the corresponding normal tissues using the TCGA and GTEx databases. (**C**–**H**) The relationship between IL18RAP expression level and pathological stages of COAD (**C**), KIRP (**D**), LUAD (**E**), SKCM (**F**), TGCT (**G**), and THCA (**H**). *p < 0.05, **p < 0.01, ***p < 0.001 and ****p < 0.0001.

We learned from the TIMER2.0 database that there were significant differences between the levels of IL18RAP expression in various cancers and the corresponding normal tissues (Supplementary Figure 1A). For further exploration, using GTEx and TCGA databases, we downloaded the RNA sequencing data across 33 types of human cancers and corresponding paracancerous tissues. First, we discovered that, when compared to normal tissues, the 13 tumors had significantly different levels of IL18RAP expression based on TCGA data. Among them, kidney renal clear cell carcinoma (KIRC), kidney chromophobe (KICH), head and neck squamous cell carcinoma (HNSC), and glioblastoma multiforme (GBM) had higher levels of IL18RAP mRNA expression than normal tissues did. The IL18RAP mRNA levels, on the other hand, were downregulated in several cancer types, including thyroid carcinoma (THCA), rectum adenocarcinoma (READ), pancreatic adenocarcinoma (PAAD), lung squamous cell carcinoma (LUSC), lung adenocarcinoma (LUAD), liver hepatocellular carcinoma (LIHC), colon adenocarcinoma (COAD), and bladder urothelial cancer (BLCA) (Figure 1A). The TCGA database mainly contains information on tumor samples and a few normal tissue data. Thus, we again analyzed the mRNA expression levels of IL18RAP in pan-cancer based on GTEx and TCGA databases. According to the findings, IL18RAP mRNA levels were increased in the GBM, HNSC, KIRC, PAAD, and testicular germ cell tumors (TGCT). Nevertheless, the expression of IL18RAP mRNA was downregulated in the following cancers: uterine carcinosarcoma (UCS), uterine corpus endometrial carcinoma (UCEC), esophageal carcinoma (ESCA), thymoma (THYM), THCA, breast invasive carcinoma (BRCA), READ, stomach adenocarcinoma (STAD), skin cutaneous melanoma (SKCM), COAD, LUAD, LIHC, prostate adenocarcinoma (PRAD), ovarian serous cystadenocarcinoma (OV), LUSC, brain lower grade glioma (LGG), acute myeloid leukemia (LAML), BLCA, KICH, lymphoid neoplasm diffuse large B-cell lymphoma (DLBC), and adrenocortical carcinoma (ACC) (Figure 1B). Subsequently, we used Sangerbox to analyze the tumor stage data and visualize the results. And the findings revealed that the stage of THCA, TGCT, SKCM, LUAD, kidney renal papillary cell carcinoma (KIRP), and COAD was connected to the expression of IL18RAP. (Figure 1C–1H).

### The diagnostic and prognostic value of IL18RAP across cancers

First, we used the “pROC” and “ggplot2” R tools to examine the diagnostic value of IL18RAP in a variety of cancers. As shown in Supplementary Figure 1B, the 12 types of cancer with the highest area under curve (AUC) value were selected. These 12 types of cancer were ACC, BRCA, COADREAD, DLBC, KICH, KIRC, LAML, LUADLUSC, OV, TGCT, THYM, and UCS.

**Figure 2:**
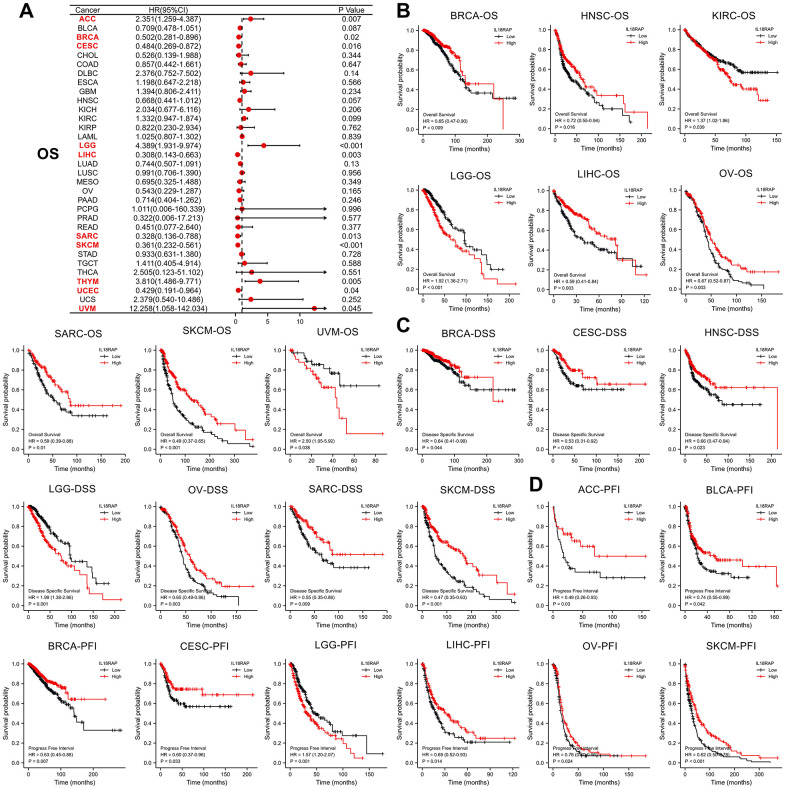
**The prognostic value of IL18RAP in pan-cancer.** (**A**) the relationship between OS and IL18RAP mRNA level in different cancers. (**B**) The relationship between the IL18RAP expression and OS in 9 cancers. (**C**) The relationship between the IL18RAP expression and DSS in 7 cancers. (**D**) The significant relationship between the IL18RAP expression and PFI in 8 cancers. All analyses were based on TCGA database.

Following that, we investigated the relationship between the mRNA expression levels of IL18RAP and overall survival (OS) in 33 different types of human cancers using single variate Cox regression analysis. Uveal melanoma (UVM), UCEC, THYM, SKCM, sarcoma (SARC), LIHC, LGG, cervical squamous cell carcinoma and endocervical adenocarcinoma (CESC), BRCA, and ACC all demonstrated significant hazard ratios (HRs) for IL18RAP, with UVM having the highest HR (12.258) (Figure 2A). Additionally, the relationship between the levels of IL18RAP expression and OS, progression free interval (PFI), and disease specific survival (DSS) in various cancers was investigated using Kaplan-Meier analysis. The results suggested that in BRCA, HNSC, LIHC, OV, SARC and SKCM, high IL18RAP groups had statistically better OS than the low IL18RAP groups. However, the high IL18RAP groups showed statistically worse OS than the low IL18RAP groups in KIRC, LGG and UVM (Figure 2B). For DSS analysis, the results showed that IL18RAP played a risk role for LGG and a protective role for SKCM, SARC, OV, HNSC, CESC, and BRCA (Figure 2C). For PFI analysis, the results showed that IL18RAP played a risk role for LGG and a protective role for SKCM, OV, LIHC, CESC, BRCA, BLCA, and ACC (Figure 2D). These findings demonstrate that higher levels of IL18RAP expression are associated with better patient survival and prognostic indicators in the majority of cancers.

### IL18RAP genetic alteration and DNA methylation in pan-cancer

**Figure 3:**
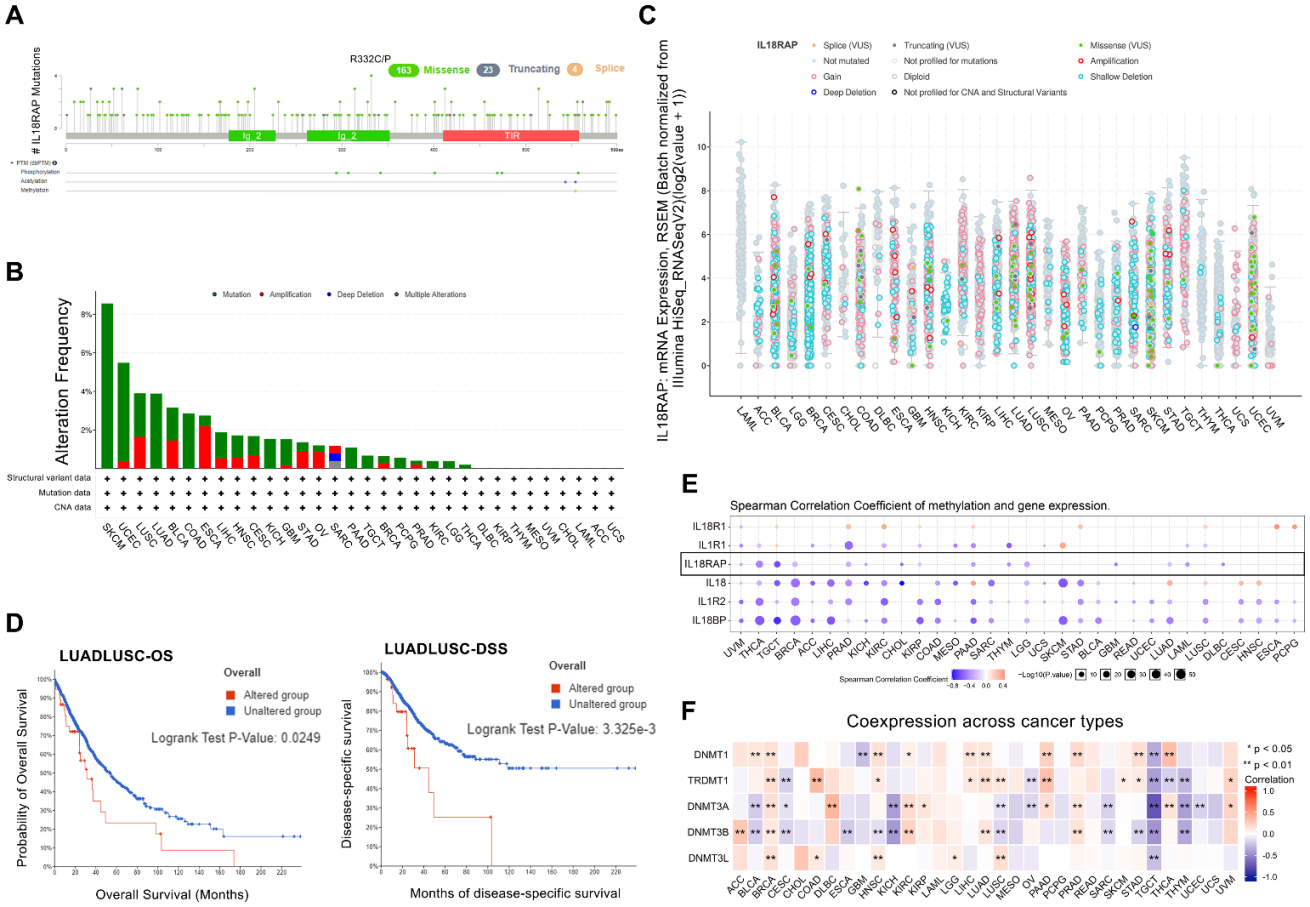
**The genetic alteration and DNA modification character of IL18RAP.** (**A**) The frequency and types of IL18RAP somatic mutations in pan-cancer. (**B**) Alteration frequency of IL18RAP in pan-cancer. (**C**) The counts and types of IL18RAP mutation in pan-cancer. (**D**) OS and DSS analysis of LUADLUSC stratified by IL18RAP alteration status. (**E**) The relationship between IL18RAP expression and DNA methylation in pan-cancer was discovered using the GSCALite database. The results of IL18RAP were circled in the black box. (**F**) The relationship between IL18RAP expression and five methyltransferases in 33 different types of human cancers was examined using the TCGA database. *p < 0.05 and **p < 0.01.

Using the cBioPortal platform, we investigated the IL18RAP alteration sites, alteration frequency, and alteration type across cancers. As shown in Figure 3B, mutation was the most frequent alteration of IL18RAP in SKCM (>8%). Figure 3A, 3C demonstrated the locations and types of IL18RAP genetic changes, with missense mutation being the most common kind. Through the GSCALite platform, we found that the mutation rate of IL18RAP reached 39% in 357 samples (Supplementary Figure 2A), and we also observed an apparent heterozygous amplification and deletion of IL18RAP in pan-cancer (Supplementary Figure 2B). We also investigated at whether the IL18RAP change would impact the prognosis of cancer patients. As shown in Figure 3D, the results confirmed that LUADLUSC patients in the IL18RAP-altered group had poorer prognoses in terms of OS and DSS.

We next investigated the relationship between DNA methylation and IL18RAP expression in human cancers utilizing the GSCALite platform since DNA methylation frequently impacts gene expression and cancer prognosis. The results demonstrated that the IL18RAP expression levels and DNA methylation were negatively correlated in 15 cancers, including UVM, THCA, TGCT, BRCA, PRAD, KICH, cholangiocarcinoma (CHOL), KIRP, PAAD, THYM, LGG, GBM, LUAD, LAML, and DLBC (Figure 3E). Additionally, we explored the correlation between IL18RAP and five methyltransferases (DNMT3L, DNMT3B, DNMT3A, TRDMT1, and DNMT1) across cancers. The findings showed that in 27 tumors, IL18RAP was associated with at least one of the five methyltransferases (Figure 3F). Among them, IL18RAP is positively correlated with all five methyltransferases in BRCA. In contrast, IL18RAP was negatively correlated with all five methyltransferases in TGCT. Nevertheless, more research is still required to determine the precise impacts of DNA methylation on the IL18RAP levels in these cancers.

### IL18RAP is associated with TMB, MSI and immune checkpoint genes in pan-cancer

**Figure 4:**
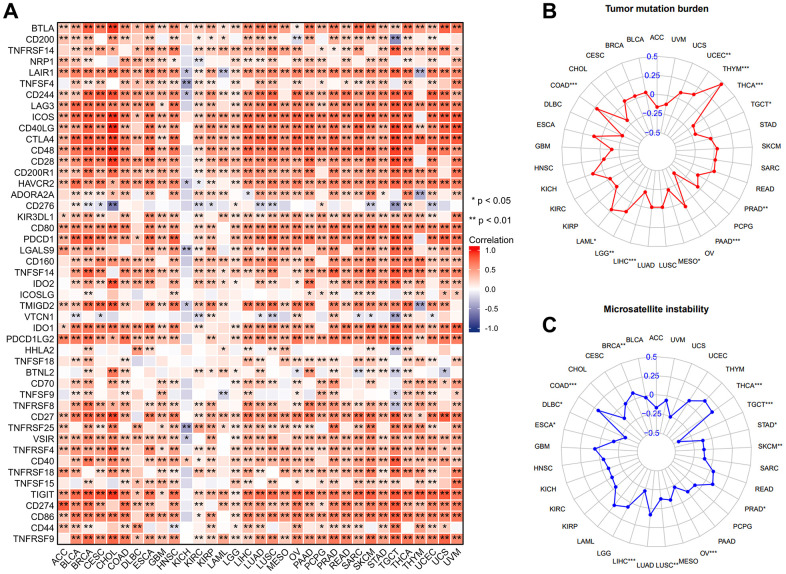
**The correlation between IL18RAP expression and immune checkpoint genes, TMB, MSI in pan-cancer.** (**A**) Excluding KICH, the expression of IL18RAP was closely correlated with immune checkpoint genes in 32 cancers except KICH. (**B**) The analysis's findings on the relationship between TMB and IL18RAP mRNA level. (**C**) The analysis's findings on the relationship between MSI and IL18RAP mRNA level. *p < 0.05 and **p < 0.01.

TMB and MSI are well-known characteristics of the TME and are considered to be involved in tumor mutation and epigenetic alterations. Figure 4B demonstrated that IL18RAP was inversely correlated with TMB in LIHC, mesothelioma (MESO), PAAD, PRAD, TGCT, and THCA, and positively correlated with TMB in COAD, LAML, LGG, THYM, and UCEC. In terms of the relationship between MSI and IL18RAP, we found that TGCT, STAD, SKCM, OV, LUSC, LIHC, ESCA, and DLBC exhibited a negative relationship, while THCA, PRAD, COAD, and BRCA exhibited a positive relationship (Figure 4C). In conclusion, our analysis suggested that IL18RAP may affect antitumor immunity by regulating the mutation and epigenetic status of TME. We then investigated at the connections between IL18RAP and 47 known immune checkpoint genes because it is generally recognized that immune checkpoint genes are crucial in tumor escape from immune destruction. The analysis’s findings revealed that, with the exception of KICH, the majority of immune checkpoint genes in 32 human cancers were positively correlated with IL18RAP, suggesting that IL18RAP may be capable of regulating tumor immunity in these cancers (Figure 4A). In addition, our research also explored the correlation between IL18RAP and MHC molecules, immunostimulators, chemokines, and chemokine receptors. As shown in Supplementary Figure 3A–3D, IL18RAP and most genes were positively correlated in 32 cancers except KICH.

### Correlation between IL18RAP expression and the TME and immune cell infiltration in pan-cancer

**Figure 5:**
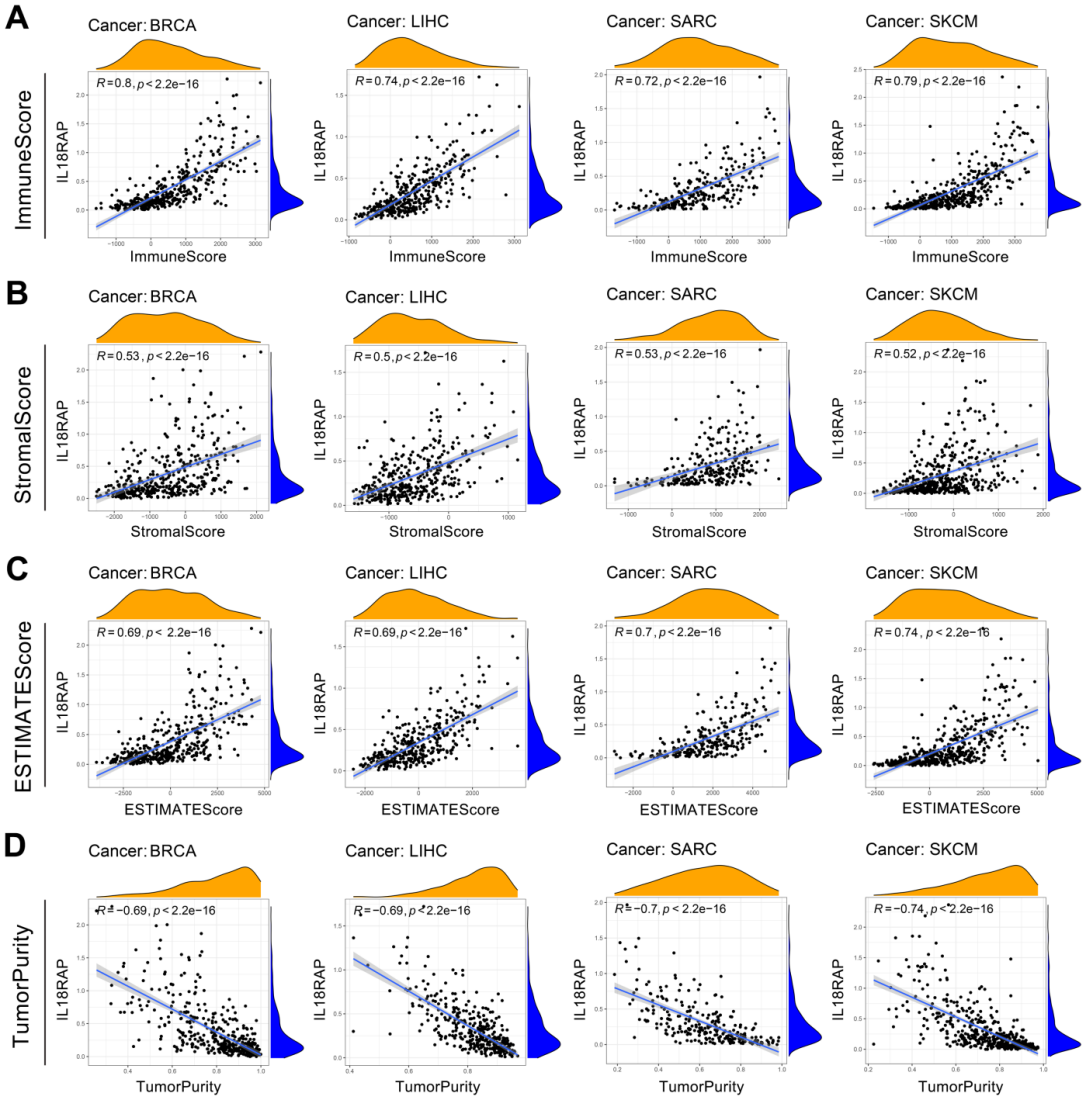
**The relevance of IL18RAP Expression to the TME.** (**A**–**D**) The relationship between IL18RAP mRNA expression level and immune score (**A**), stromal score (**B**), ESTIMATE score (**C**), and tumor purity (**D**) in BRCA, LIHC, SARC, and SKCM is shown in the graph. The analyses were based on TCGA database.

Immune cells and stromal cells are two significant components of TME, and it is widely recognized that the TME plays a significant role in the cancer occurrence and development. We assessed the ESTIMATE score, tumor purity, immune score, and stromal score across 33 different types of human cancers using the “ESTIMATE” R package. As shown in Figure 5, the four cancers (BRCA, LIHC, SARC, SKCM) with the highest ESTIMATE score were presented, and SKCM had the highest ESTIMATE score (R=0.74). For most cancers, IL18RAP was positively linked to the immune and stromal score and negatively linked to tumor purity, indicating that IL18RAP may affect tumor progression by encouraging stromal and immune cell infiltration in the TME (Figure 5A–5D).

**Figure 6:**
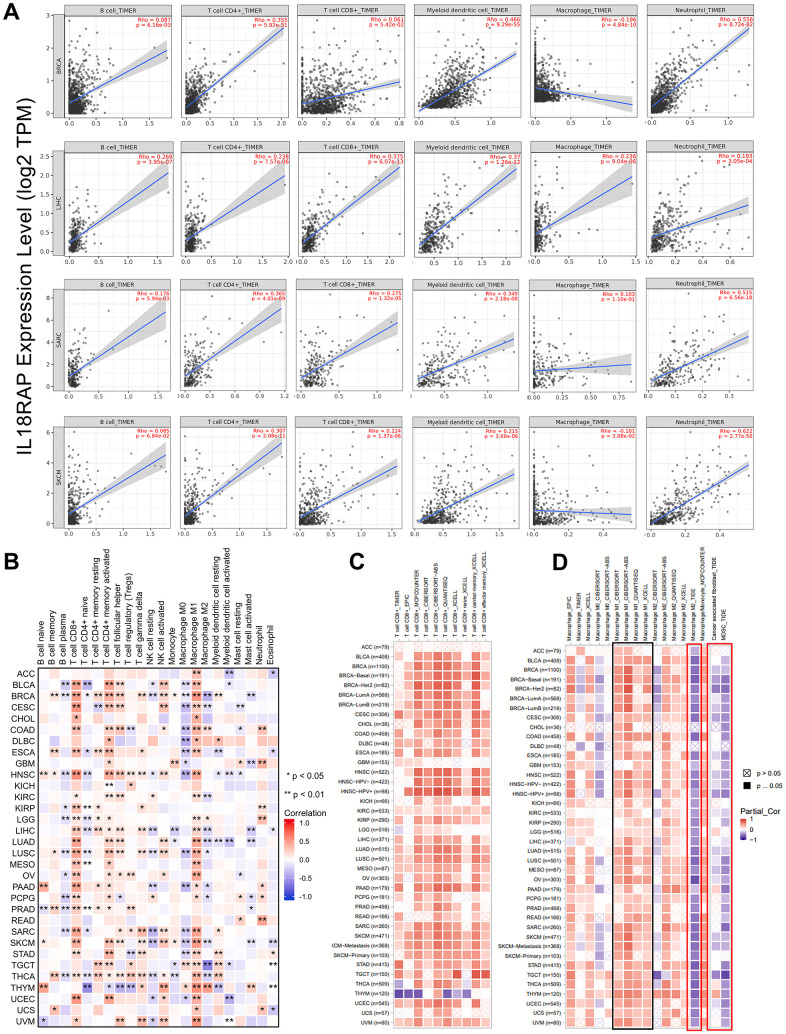
**The correlation analysis of IL18RAP expression and immune cells infiltration in pan-cancer.** (**A**) The relationship between mRNA expression level of IL18RAP and infiltration of CD4+T cells, B cells, macrophages, CD8+T cells, dendritic cells and neutrophils in BRCA, LIHC, SARC, and SKCM was examed using TIMER algorithm. (**B**) Using the CIBERSORT algorithm, the relationship between IL18RAP expression and the infiltration of 22 different immune cell types in pan-cancer was determined. (**C**, **D**). Correlation of IL18RAP expression with the infiltration of CD8+T cells (**C**) and different kinds of macrophages (**D**) obtained from TIMER2.0 database. The results of M1 macrophages were circled in the black box. The results of M2 macrophages, CAFs, and MDSCs were circled in the red box.

The TIMER2.0 database was then analyzed in order to further assess the relationship of IL18RAP expression and immune cells across cancers. Using the TIMER algorithm, we were able to determine the correlations between the mRNA expression of IL18RAP and B cells, CD4+ T cells, CD8+ T cells, macrophages, neutrophils, and dendritic cells in BRCA, SARC, and SKCM. As shown in Figure 6A, except for macrophages, IL18RAP showed strong positively correlations with the other five kinds of immune cells. In addition, the relationship between 22 different types of immune cells and IL18RAP expression across cancers was determined using the CIBERSORT algorithm. Among which, IL18RAP was associated with activated CD4+ memory T cells in 24 cancer types, CD8+ T cells in 24 cancer types, and M1 macrophages in 27 cancer types in a positive correlation (Figure 6B). Interestingly, we observed that IL18RAP is negatively related to M0 macrophages and M2 macrophages in various cancers, which may be the reason why the TIMER algorithm failed to count the correlation between IL18RAP and macrophages because the TIMER algorithm does not analyze macrophage subtypes. The connection between IL18RAP expression and CD8+ T cells and macrophages was calculated using a variety of algorithms. The findings supported the previous finding that IL18RAP expression was positively associated with CD8+ T cells and M1 macrophages in a number of cancers (Figure 6C, 6D). Moreover, we also observed that IL18RAP expression was significantly negatively related to cancer-associated fibroblast and myeloid-derived suppressor cell infiltration levels in most cancers (Figure 6D). In summary, IL18RAP can enhance the infiltration of multiple immune cells in a variety of cancers, thereby inhibiting tumor progression.

### Single-cell RNA sequencing analysis of IL18RAP

**Figure 7:**
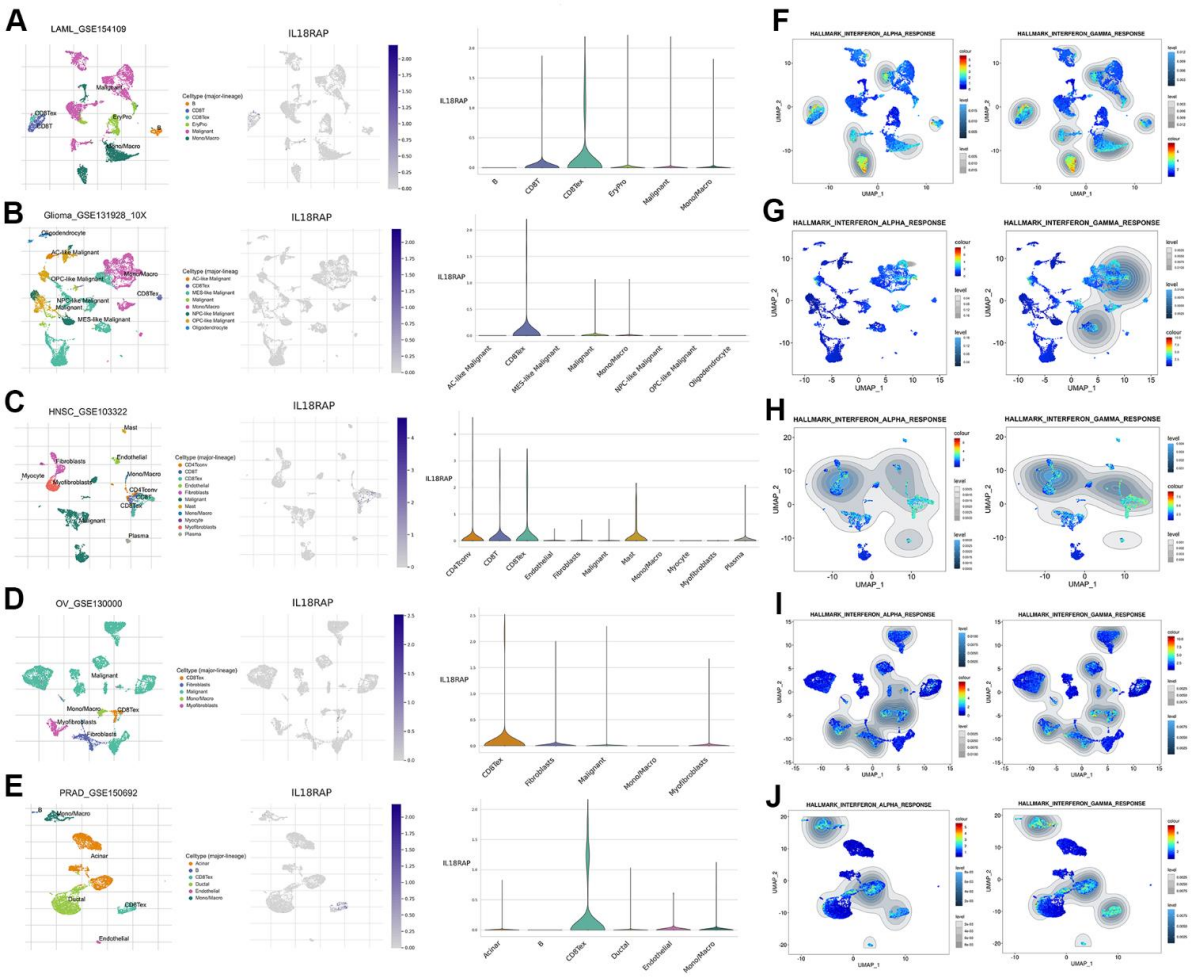
**Single-cell RNA sequencing analysis of IL18RAP.** (**A**–**E**) The definition of cell subtypes and the analysis of IL18RAP expression in different clusters of cells in LAML, Glioma, HNSC, OV, and PRAD. (**F**–**J**) The single-cell signature analysis of CD8+T cells in LAML, Glioma, HNSC, OV, and PRAD. The analysis was performed on TISCH2 database.

Through the TISCH2 database, we identified the cell subtypes in LAML (GSE154109), glioma (GSE131928_10X), HNSC (GSE103322), OV (GSE130000), and PRAD (GSE150692) and described the IL18RAP expression levels in different clusters of cells (Figure 7A–7E). IL18RAP was shown to be enriched in NK cells, CD8+ T cells, and CD8Tex cells, according to the analysis’s findings. Among them, CD8Tex cells caught our attention. Through the “GSEA” section of the “Dataset” module, we analyzed the single-cell signature of these cell clusters and found that CD8Tex cells were closely related to INF-a and INF-γ (Figure 7F–7J). Subsequently, we performed HALLMARK gene set analysis on these cell clusters, and the results confirmed that INF-a and INF-γ response gene sets were significantly upregulated in CD8Tex cells (Supplementary Figure 4A–4D). Combined with the previous GSEA results and immunotherapy response results, it is reasonable to assume that the complex interactions between IL18RAP, CD8Tex cells, and INF-γ plays a critical role in regulating TME.

### The role of IL18RAP in the recruitment of M1 macrophages

**Figure 8:**
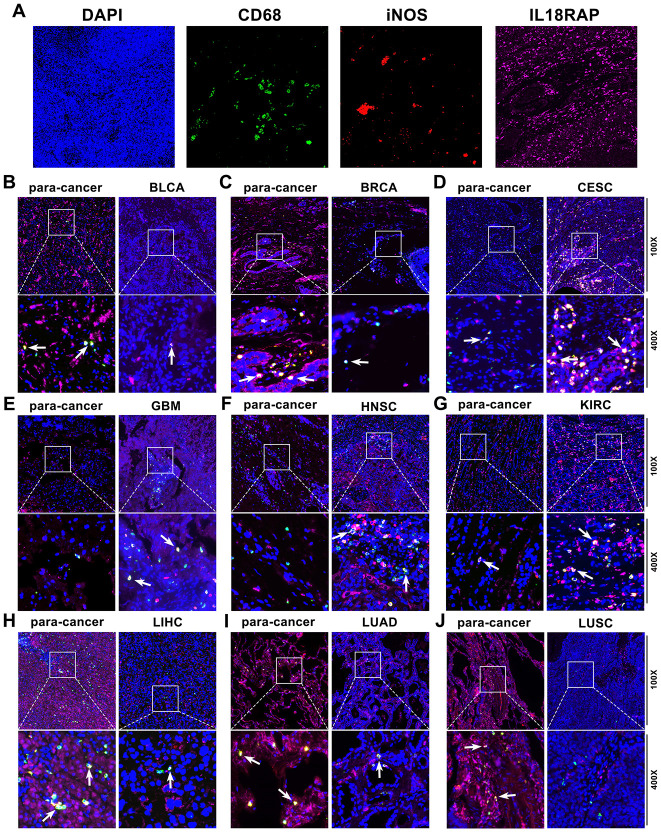
**The expression of IL18RAP, CD68, and iNOS in 9 cancers and the corresponding paracancerous tissues detected by multiplex immunofluorescence staining.** (**A**) The representative image of DAPI, CD68, iNOS, and IL18RAP, respectively. Blue represents the DAPI-stained nucleus; red represents CD68-positive cells; green represents iNOS-positive cells; and pink represents IL18RAP-positive area. (**B**–**J**) The representative immunofluorescence images of BLCA (**B**), BRCA (**C**), CESC (**D**), GBM (**E**), HNSC (**F**), KIRC (**G**), LIHC (**H**), LUAD (**I**), LUSC (**J**) and corresponding para-cancerous tissues. The white arrow indicates CD68 and iNOS double-positive cells.

**Figure 9:**
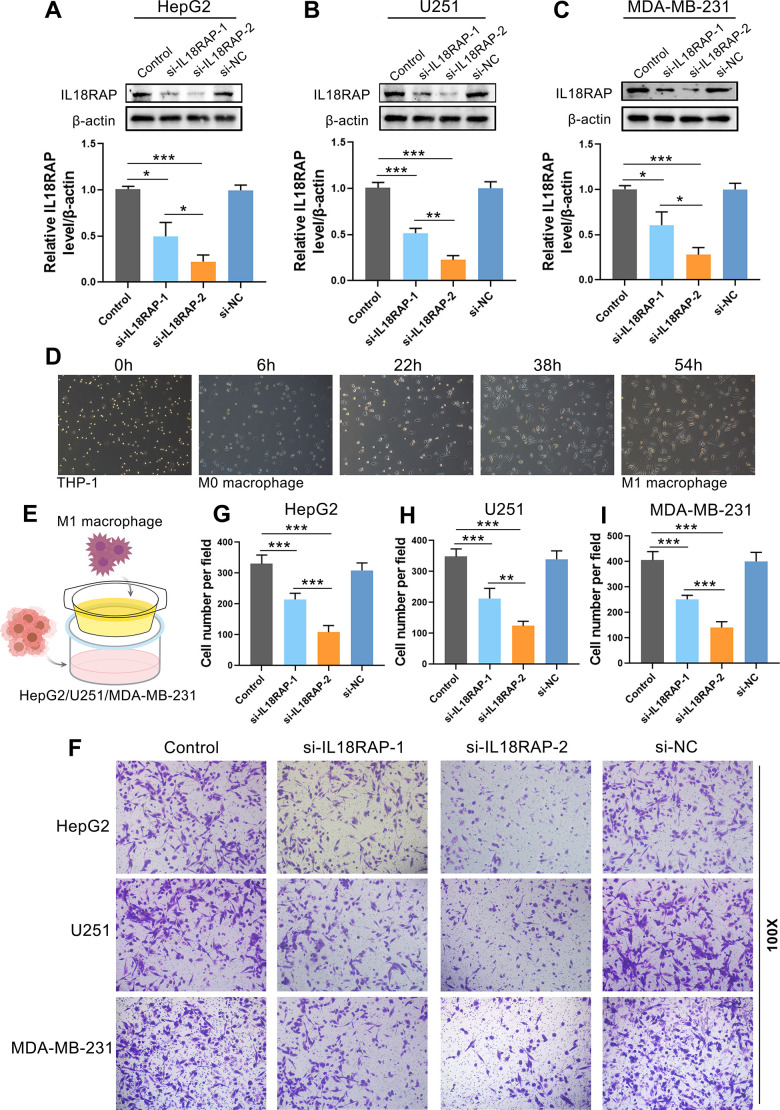
**Downregulation of IL18RAP reduced the migration ability of M1 macrophages.** (**A**–**C**) After transfection of HepG2, U251 and MDA-MB-231 with different siRNA, the IL18RAP protein levels was detected. (**D**) The induction process and morphology of M1 macrophages. (**E**) The schematic representation of the coculture of different cells. (**F**–**I**) The M1 macrophages’ migration after being cocultured with HepG2, U251 and MDA-MB-231 cells that have been transfected with si-IL18RAP-1, si-IL18RAP-2 or si-NC. *p < 0.05, **p < 0.01, ***p < 0.001.

Based on the results of the aforementioned bioinformatics investigation, we have known that IL18RAP expression is positively correlated with the infiltration of various immune cells, especially M1 macrophages. In order to determine whether IL18RAP expression in cancer cells is crucial for the infiltration of M1 macrophages, immunofluorescence staining and *in vitro* experiments were used for further investigation. Multiple immunofluorescence staining was utilized to identify the IL18RAP expression and the M1 macrophage markers CD68 and iNOS in BRCA, BLCA, GBM, CESC, KIRC, HNSC, LUAD, LIHC, and LUSC. According to the findings, which are shown in Figure 8, IL18RAP was increased in CESC, GBM, HNSC, and KIRC while downregulated in BLCA, BRCA, LIHC, LUAD, and LUSC when compared to the corresponding paracancerous tissues. This result was consistent with our previous findings in this article. Moreover, we found a positive relationship between the amount of M1 macrophages (CD68 and iNOS double- positive cells) and the IL18RAP level, suggesting that IL18RAP may be crucial for the recruitment of M1 macrophages in the TME (Figure 8B–8J). Subsequently, we attempted to explore further *in vitro* experiments. The human GBM cell Line U251, the BRCA cell line MDA-MB-231 and the LIHC cell Line HepG2 was transfected with si-IL18RAP-1, si-IL18RAP-2 or si-NC. In contrast to the control group and the si-NC group, Western blotting demonstrated that transfection of si-IL18RAP-1 and si-IL18RAP-2 led in a considerable reduction of IL18RAP protein level (Figure 9A–9C). Then, we stimulated the differentiation of human THP-1 cells into M1 macrophages *in vitro* (Figure 9D). On this basis, M1 macrophages were co-cultured with U251, MDA-MB-231, and HepG2 cells transfected with different kinds of siRNA through a Transwell apparatus, and the effect of IL18RAP on M1 macrophages migration ability was calculated by counting the number of cells crossing the upper chamber (Figure 9E). The results revealed that IL18RAP knockdown in U251, MDA-MB-231 and HepG2 cells significantly inhibited the migration ability of M1 macrophages in coculture experiments (Figure 9F). In summary, the above studies confirmed that high levels of IL18RAP can promote the chemotaxis of M1 macrophages in TME.

### PPI and functional enrichment analysis of IL18RAP in cancers

**Figure 10:**
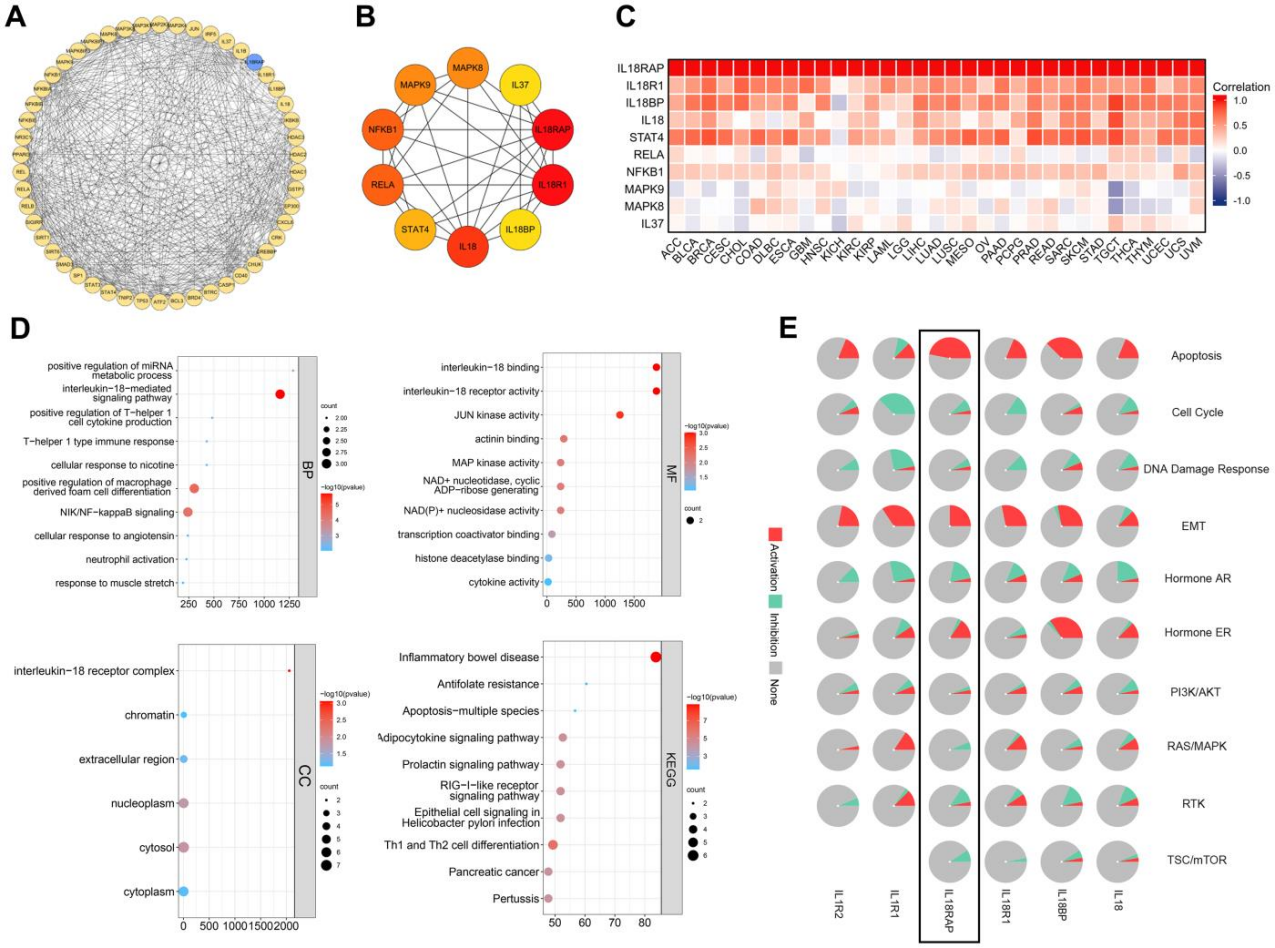
**The construction of the PPI network and an analysis of IL18RAP's functional enrichment in cancers.** (**A**, **B**) The PPI network (**A**) of IL18RAP constructed by Cytoscape, and the top 10 hub genes (**B**) of PPI were selected using cytoHubba plugins. (**C**) The correlation between the top 10 hub genes with IL18RAP in 33 types of cancers was calculated using Spearman correlation analysis. (**D**) The GO/KEGG enrichment analyses of the top 10 hub genes. (**E**) The relationship between IL18RAP and 10 famous cancer-related pathways was analyzed via GSCALite platform. The results of IL18RAP were circled in the black box.

First, through the STRING database, 50 genes closely related to IL18RAP were acquired and a Protein-Protein Interaction (PPI) network was constructed using Cytoscape (Figure 10A). On this basis, we used the cytoHubba plugins to extract the top 10 related genes and calculated their correlation with IL18RAP in 33 types of cancers using Spearman correlation analysis (Figure 10B, 10C). These top 10 hub genes were IL18, IL18RAP, IL18R1, IL18BP, STAT4, RELA, NFKB1, MAPK9, MAPK8, and IL37. Following that, investigations of KEGG/GO enrichment were carried out on the top 10 genes (Figure 10D). The top 5 GO terms of Biological Process (BP) were interleukin-18-mediated signaling pathway, positive regulation of miRNA metabolic process, positive regulation of T-helper 1 cells cytokine production, cellular response to nicotine, T-helper 1 type immune response; Cellular Component (CC) were nterleukin-18 receptor complex, chromatin, extracellular region, nucleoplasm, cytosol; Molecular Function (MF) were interleukin-18 binding, interleukin-18 receptor activity, JUN kinase activity, actinin binding, MAP kinase activity. The top 5 KEGG pathways were Inflammatory bowel disease, Antifolate resistance, Apoptosis-multiple species, Adipocytokine signaling pathway, Prolactin signaling pathway. By using the GSCALite database, we explored the correlation between IL18RAP and well-known cancer-related pathways activated or inhibited across cancers. The results showed that the pathways activated by IL18RAP were mainly apoptosis, EMT, and ER, and those inhibited were TSC/mTOR, RTK and so on (Figure 10E).

**Figure 11:**
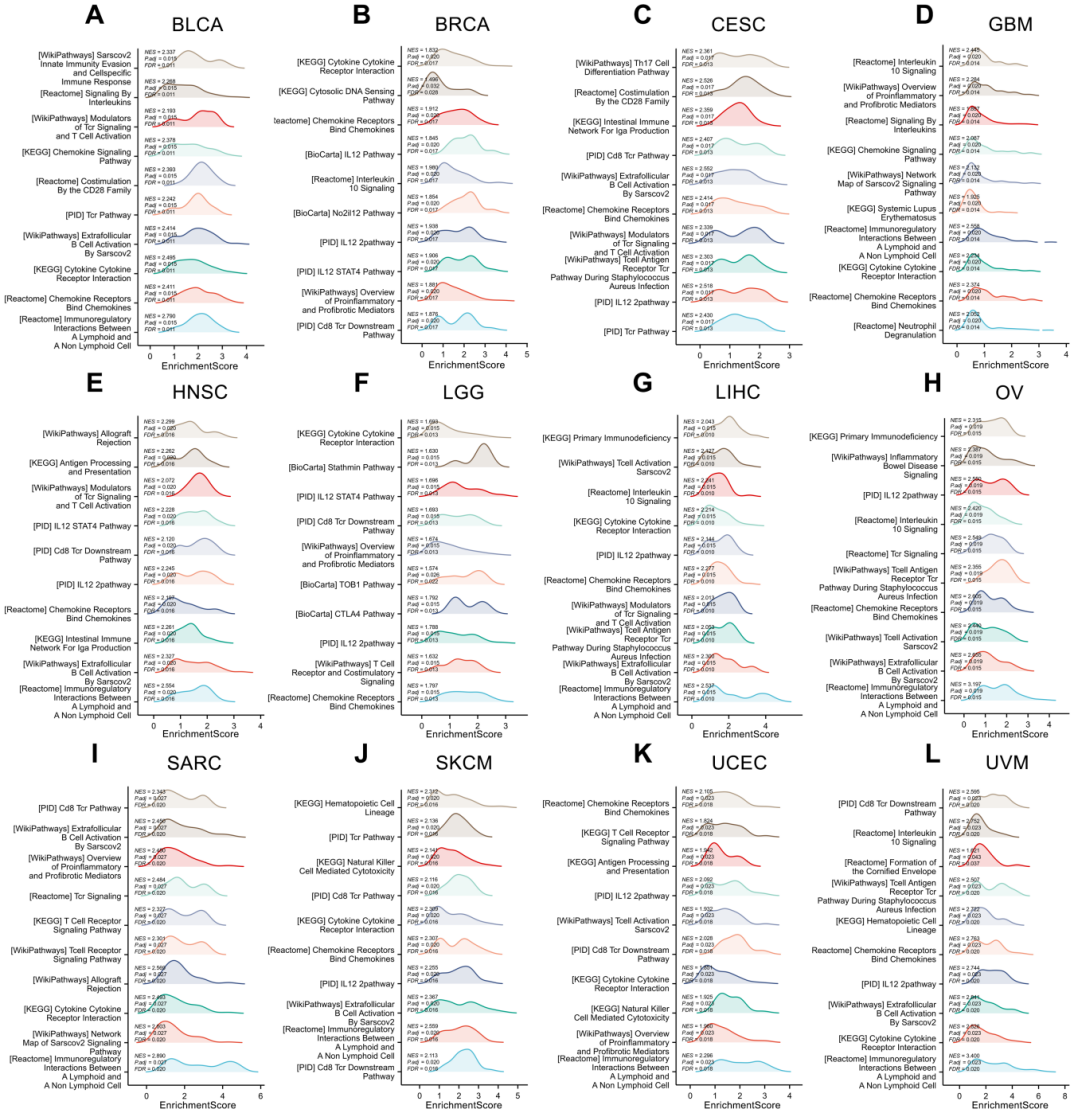
**Gene set enrichment analysis of IL18RAP in 12 cancers.** (**A**–**L**) The GSEA functional enrichment analysis of IL18RAP in BLCA (**A**), BRCA (**B**), CESC (**C**), GBM (**D**), HNSC (**E**), LGG (**F**), LIHC (**G**), OV (**H**), SARC (**I**), SKCM (**J**), UCEC (**K**), and UVM (**L**). The analyses were based on TCGA database.

The GSEA analysis results of IL18RAP in 12 cancers are shown in Figure 11A–11L. Statistics show that common enrichment pathways are chemokine signaling pathway, chemokine receptors bind chemokines, IL12 2 pathway, IL12 STAT4 pathway, T cell receptor signaling pathway and so on. The above analysis results indicate that IL-18RAP is widely involved in activities such as immune cell activation, chemokine function activation, IL12 Signaling pathway transduction in a variety of cancers.

### Immunotherapy response and drug sensitivity analysis of IL18RAP

**Figure 12:**
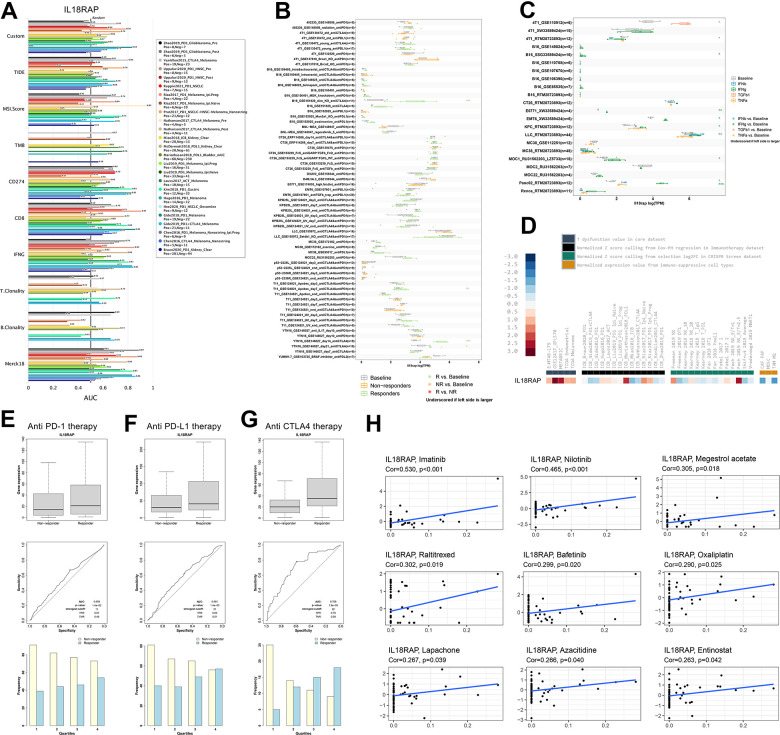
**Immunotherapy response and drug sensitivity analysis of IL18RAP.** (**A**) Ability of the IL18RAP to predict OS and response outcomes in cohorts of patients receiving immunotherapy. (**B**) The TISMO database was used to investigate the predictive power of IL18RAP in mouse immunotherapy cohorts. (**C**) The TISMO database was used to examine the expression levels of IL18RAP in cell lines that had undergone different treatments. (**D**) The IL18RAP expression in various datasets obtained from the TIDE platform. (**E**–**G**) The effectiveness of IL18RAP as a predictor in response to anti-PD-1 (**E**), anti-PD-L1 (**F**), and anti-CTLA4 (**G**) therapy. (**H**) The relationship between IL18RAP expression and drug sensitivity of 9 common anticancer drugs.

Through TIDE database, we explored the predictive power of OS and response outcomes response of IL18RAP as a biomarker for the human immunotherapy cohort. As shown in Figure 12A, the AUC of IL18RAP was greater than 0.5 in 16 of 25 immunotherapy cohorts. The capacity of IL18RAP to predict outcomes is superior to that of TMB, MSI Score, B. clonality, T. clonality, and as can be seen through comparison. However, IL18RAP’s predictive power is inferior to that of TIDE, CD8, CD274, Merck18, and IFNG.

Through the TISMO database, we discovered that IL18RAP exhibits a great ability to predict the immunotherapy response in 4 murine immunotherapy cohorts. Interestingly, these four cohorts all included anti-CTLA4 therapy, and the level of IL-18RAP in the responders increased significantly (Figure 12B). This tends to indicate that IL18RAP and the anti-CTLA4 therapeutic effect are closely connected, although more experimental proof is required. In addition, the expression levels of mRNA of IL18RAP across cell lines in the pre- and post-cytokine-treated groups are shown in Figure 12C. Notably, INF-γ treatment significantly increased the expression level of IL18RAP in the seven control groups. This suggests an important relationship between IL18RAP and INF-γ, which is consistent with the GSEA results of IL18RAP. It is apparent from comparing the expression levels of IL18RAP across several datasets that the expression of IL18RAP was markedly elevated in the core dataset. Contrarily, most of the IL18RAP levels in the CRISPR screen dataset and immunosuppressive cell types decreased (Figure 12D). Further research revealed that responders who had received anti-CTLA4, anti-PD-L1, and anti-PD-1 therapy had their expression of IL18RAP considerably raised (Figure 12E–12G). Among them, the anti-CTLA4 therapy group had the highest AUC (0.789) (Figure 12G). Furthermore, the CellMiner database was used to retrieve all the drug sensitivity data, and R software was utilized for visualization. The findings demonstrated that in NCI-60 cell lines treated with imatinib, nilotinib, megestrol acetate, raltitrexed, bafetinib, oxaliplatin, lapachone, azacitidine and entinostat, the mRNA expression level of IL18RAP was positively correlated with the therapeutic response (Figure 12H). This suggested that the IL18RAP level could reflect the sensitivity of multiple cancer cell lines to anti-cancer drugs. In addition, in the CTRP database, low levels of IL18RAP were found to be related to increased drug resistance to many drugs (Supplementary Figure 5A). In the GDSC database, we found that low levels of IL18RAP were associated with increased drug resistance to MPS-1-IN-1, CH5424802, XMD14-99, TPCA-1, XMD15-27, and KIN001-260 (Supplementary Figure 5B). The results above conclude that the expression level of IL18RAP may influence the sensitivity to some common chemotherapeutic medications as well as the responsiveness to various immunotherapies.

### IL18RAP knockdown promoted the proliferation, migration and invasion of MDA-MB-231 cells

**Figure 13:**
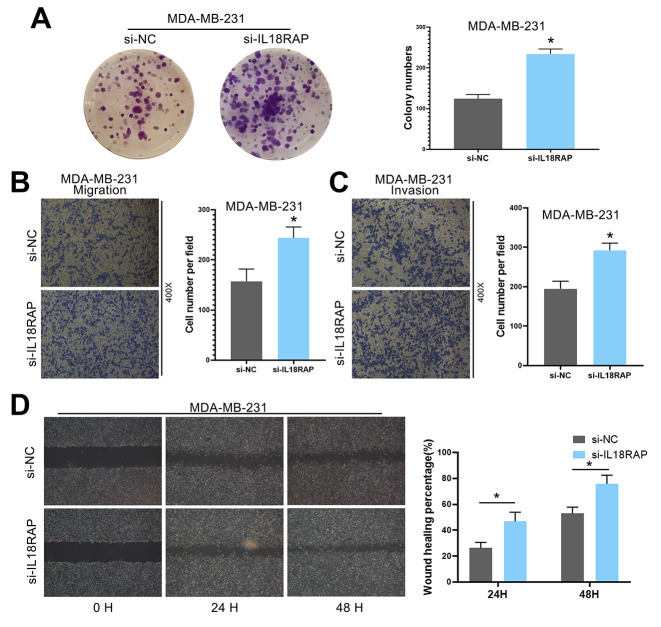
**Downregulation of IL18RAP enhanced the proliferation, migration and invasion of MDA-MB-231 cells.** (**A**) Si-IL18RAP enhanced the colony formation ability of MDA-MB-231 cells. (**B**, **C**) The migration and invasion of MDA-MB-231 cells were enhanced after transfection with si-IL18RAP as measured by a Transwell assay. (**D**) The migration of MDA-MB-231 cells was enhanced after transfection with si-IL18RAP as measured by a wound-healing assay. *p < 0.05.

To explore the effect of IL18RAP on breast cancer cells MDA-MB-231, MDA-MB-231 was transfected with siRNA and *in vitro* studies were performed. As expected, the results showed that MDA-MB-231 cells in the si-IL18RAP group proliferated faster (Figure 13A). Transwell assay and wound-healing assay also confirmed that the migration and invasion ability of MDA-MB-231 cells were enhanced after the down-regulation of IL18RAP level (Figure 13B–13D). In summary, these results indicate that MDA-MB-231 cells expressing lower levels of IL18RAP exhibit greater proliferation, migration, and invasion abilities.

## DISCUSSION

IL18 is considered to be an inflammatory factor which plays a crucial immune regulation role in cancers [20–22]. The significance of IL18RAP in cancers has received little research despite being a significant factor of IL18 signaling. Therefore, it is essential and meaningful to conduct a comprehensive pan-cancer analysis to reveal the IL18RAP’s potential biological functions in various cancers. In this research, we identified the IL18RAP mRNA expression level, clinical characteristics, and genetic alterations in cancers. Additionally, we investigated the relationships between IL18RAP and immune infiltration, TMB, MSI, and immune checkpoint genes. Through further exploration, this study clarified the biological function of IL18RAP across cancers and its ability to predict the immunotherapy response.

We discovered that IL18RAP was differentially expressed in various cancers, and the IL18RAP level was downregulated significantly in most cancers and correlated with clinical stage. ROC curve results suggested that IL18RAP may be a potential new diagnostic biomarker. The study of prognostic data demonstrated that downregulation of IL18RAP was related to poor OS in BRCA, CESC, HNSC, LIHC, OV, SARC, SKCM, and UCEC, which was also verified by Zhuang et al. in LIHC [23]. Additionally, low IL18RAP expression was related to worse DSS and PFI in a variety of cancers. Although we found that low levels of IL18RAP in MDA-MB-231 cells could enhance the proliferation, migration and invasion abilities, no research has yet explored the precise function of IL18RAP in these cancers.

Increasing evidence has demonstrated that genetic alteration and DNA modifications can significantly affect gene expression in cancers [24, 25]. Our analysis showed that copy number variation (CNV) and DNA methylation were significantly related to IL18RAP expression levels. DNA methylation has been shown to significantly reduce gene levels. This study revealed a negative correlation between DNA methylation and IL18RAP, which may account for the low levels of IL18RAP expression in many cancers. In addition, other open database data show that the IL18RAP mutation frequency significantly affects OS and DSS in LUADLUSC and THCA patients, although the IL18RAP mutation frequency is low in human cancers. More investigations are required to determine the effect of DNA methylation and genetic changes on IL18RAP in cancers due to the lack of IL18RAP research in this area.

In recent years, cancer immunotherapy has made breakthrough progress, and the TME is becoming a hot spot in the research of cancer immunity. Malignant cells, endothelial cells, fibroblasts, immune cells and stromal cells are important components of the TME and are important to the tumor immunotherapy efficacy [26, 27]. Our research shown that IL18RAP is positively related to both stromal and immune scores in cancers, suggesting that IL18RAP may enhance the infiltration of both stromal and immune cells in TME. NK cells, CD8+ T cells, and M1 macrophages are key to the killing of tumor cells *in vivo* [28, 29], and our analysis revealed that IL18RAP can promote CD8+ T cells and M1 macrophages infiltration in various cancers. This may be related to the fact that IL18 can stimulate Th1 differentiation and enhance the cytotoxicity of NK cells through IFN-γ [30]. We verified, using tissue multiple immunofluorescence staining and coculture migration analysis for M1 macrophages, that the IL18RAP expression level was positively related to the M1 macrophages infiltration. Contrarily, research has demonstrated that cell infiltration, such as cancer-associated fibroblasts (CAFs), tumor-associated macrophages (TAMs), T-regulatory cells (Tregs), and myeloid-derived suppressor cells (MDSCs), contributes to an immunosuppressive TME, which can enable tumor cells to evade immune destruction and result in a poor prognosis [31]. Among them, TAMs, especially M2 macrophages, can block T-cell differentiation and promote the recruitment of Tregs and MDSCs, ultimately promoting the construction of an immunosuppressive TME. Moreover, M2 macrophages can directly supply nutrients to tumor cells to promote tumor growth [32]. This is in line with our findings, which showed that the protective gene IL18RAP has a negative correlation with the infiltration of M2 macrophages, CAFs, and MDSCs in various cancers.

Normally, the body’s immune system is able to recognize and eliminate cancer cells, but abnormal expression of ICI genes interferes with this function [3]. Through gene correlation analysis, we validated the association between IL18RAP and known ICI genes in cancers. It has also been investigated how the expression of IL18RAP relates to MHC molecules, immunostimulators, chemokines, and chemokine receptors genes. As expected, these findings demonstrated a statistical link between IL18RAP and the majority of these genes. This suggests that IL18RAP is indeed a key gene in the regulation of tumor immunity. On the other hand, through numerous public databases, we determined that high expression of IL18RAP can predict the immune checkpoint blockade response, further confirming the relevance between IL18RAP and ICI genes. In addition, the high expression of IL18RAP can also reduce the resistance of many common anticancer drugs. These findings suggest that IL18RAP can be used as a novel target for immunotherapy.

The results of GSEA functional analysis of IL18RAP show that gene sets associated with IL18RAP are concentrated in the positive regulation of NK cell-mediated immunity and interferon gamma production, lymphocyte-mediated immunity, regulation of T-cell-mediated immunity activation and so on. This provided more evidence that IL18RAP regulates the development of cancers by promoting the synthesis of INF-γ, which regulates T cell activation and improves NK cell killing. On the other hand, IL18RAP was found to significantly activate apoptosis and ER hormones and inhibit the AR, RTK, RAS/MAPK, and TSC/mTOR signaling pathways. This further highlights the inhibitory effect of IL18RAP on cancer progression.

In the last decade, more research has been done using scRNA-seq analysis to examine the heterogeneity of TME cells in cancers [33, 34]. By using scRNA-seq analysis, the distribution of IL18RAP in various cell types was identified. We discovered that IL18RAP was expressed in a variety of immune cells, including NK cells, CD8+ T cells and CD8+ Tex cells. Meanwhile, the HALLMARK gene set analysis results confirmed that INF-a and INF-γ response gene sets were significantly upregulated in CD8Tex cells, which is consistent with the description of IL18RAP function by many scholars [14, 35, 36]. CD8+ T-cell exhaustion seriously affects the killing of CD8+ T cells on cancer cells. However, ICB treatment can save these PD-1-expressing cells from the nonresponsive and exhaustion state to resume the response to cancer cells [37]. Considering that the current description of the correlation between CD8+ T-cell exhaustion and ICB is still controversial, why high levels of IL18RAP can indicate an ICB therapeutic response remain to be explored [38–40].

In conclusion, our research offers comprehensive views of how IL18RAP affects clinical characteristics, gene alterations, gene modifications, immune infiltration, and immunotherapy in various human cancer. The results of this study demonstrate that IL18RAP might be a new immunological and prognostic biomarker, offering a new target for cancer immunotherapy.

## MATERIALS AND METHODS

### Data mining and differential expression analysis of IL18RAP

We collected the RNA sequencing data of human cancers from the GTEx and TCGA datasets, as well as clinical data including tumor-node metastasis stage and survival time, through the UCSC Xena platform (https://xenabrowser.net/datapages/). For data analysis, we used R software (version 4.2.1). The IL18RAP expression differences between cancer and paracancerous tissues were found using the “Wilcox.test” method. Using Sangerbox (http://vip.sangerbox.com/home.html) and Xiantao Academic (https://www.xiantao.love/products/apply), we then displayed the data and produced violin graphs.

### Clinical correlation analysis of IL18RAP

Using the median level of IL18RAP expression, all patients were split into two groups as the cutoff threshold (low IL18RAP expression group and high IL18RAP expression group). Then, using Kaplan-Meier curves and forest plots, we examined and displayed the effect of IL18RAP expression on OS, DSS, DFS, and PFI. The impact of IL18RAP on patient survival was assessed using the HR.

### Analysis of IL18RAP genetic alteration

We were able to obtain information about IL18RAP’s genetic alterations (including mutation sites, mutation types, and mutation counts) through cBioPortal database (https://www.cbioportal.org/). Additionally, the correlation of IL18RAP expression and the degree of DNA methylation and gene CNV were investigated using GSCA (https://bioinfo.life.hust.edu.cn/web/GSCALite/). In the GSCA website, we have additionally entered 4 genes closely related to IL18RAP function, including IL18, IL18BP, IL1R1, IL1R2 and IL18R1. Finally, the connection between the expression of IL18RAP and the five methyltransferases (DNMT1, DNMT3A, TRDMT1, DNMT3B, and DNMT3L) was assessed using the Spearman method across 33 types of cancers.

### ROC curve analysis of IL18RAP

ROC curves were used to access the diagnostic value of IL18RAP across 33 different types of human cancers. Using the “ggplot2” and “pROC” R packages, respectively, images were calculated and plotted. The accuracy of diagnosis is shown by the AUC value.

### Analysis of immune-related characteristics of IL18RAP

First, the connection between IL18RAP expression and the ESTIMATE score, tumor purity, immunological score, and stromal score across 33 different types of human cancers was calculated. Then, we retrieved the infiltration scores from the TIMER2.0 database (http://timer.compgenomics.org/). After that, we assessed the relationship between the mRNA expression level of IL18RAP and the infiltration of several immune cell types using Spearman correlation analysis. Additionally, it was examined whether the expression of IL18RAP correlated with MSI or TMB. Finally, we investigated the relationship between the mRNA expression level of IL18RAP and immunostimulators, tumor-infiltrating lymphocytes, MHC molecules, immunoinhibitors, chemokines, and chemokine receptors in the 33 different types of human cancers using the “GSVA” R package.

### Correlation analysis of IL18RAP and drug response and immunotherapy response

Briefly, the drug sensitivity data were downloaded from the CellMiner database (http://discover.nci.nih.gov/cellminer/), and we utilized the R packages “limma” and “ggpubr” to generate and visualize the results. Moreover, using the GDSC and CTRP module, a comprehensive examination of the connection between IL18RAP expression and drug sensitivity was conducted. Then, using the Spearman approach, we determined the association between the mRNA expression level of IL18RAP and drug response sensitivity. The immunotherapy response was also predicted using the TISMO database (http://tismo.cistrome.org), ROC Plotter (http://www.rocplot.org/), and TIDE database (http://tide.dfci.harvard.edu).

### Construction of PPI network of IL18RAP

The STRING database (https://string-db.org/) was used to download information about the potential protein interactions with IL18RAP, and Cytoscape was used to import all of the information (v3.8.2). Then, using the cytoHubba plugins, we displayed the top 50 nodes and top 10 nodes ranked by MCC. Additionally, we used the Spearman approach to investigate the association of the top 10 genes across cancers.

### Functional enrichment analysis of IL18RAP

The top 10 genes screened by Cytoscape included IL18, IL18RAP, IL18R1, IL18BP, STAT4, RELA, NFKB1, MAPK9, MAPK8, and IL37. The top 10 genes’ KEGG enrichment and GO function analysis findings were then retrieved from the DAVID database (https://david.ncifcrf.gov/summary.jsp). The results were visualized using BioLadder (https://www.bioladder.cn/web/#/chart/28), an online mapping platform. Additionally, using the GSCALite database (https://bioinfo.life.hust.edu.cn/web/GSCALite/), we examined the connection between IL18RAP and well-known cancer-related pathways that were either activated or inhibited across cancers.

### Gene set enrichment analysis

We obtained gene ontology sets and curated gene sets from the GSEA (https://www.gsea-msigdb.org/gsea/downloads.jsp). The biological pathway variations between the high- and low-IL18RAP groups were identified using the “clusterProfiler” program. False discovery rate (FDR) < 0.25 and an adjusted p-value < 0.05 were regarded as remarkably modified pathways. For each analysis, the Gene set permutation should be run 1,000 times. Finally, Xiantao Academic (https://www.xiantao.love/products/apply) was used to display the results.

### Analysis of single-cell RNA sequencing

Briefly, we analyzed the correlation of IL18RAP expression and various cell types in a variety of cancers using the TISCH2 website (http://tisch.comp-genomics.org/home/). In addition, through the “GSEA” section of the “Dataset” module, we also obtained hallmark and single-cell signature analysis results for different cell types.

### Cell culture

The HepG2 (LIHC cell line), MDA-MB-231 (BRCA cell line) and U251 (GBM cell line) were cultured in DMEM. In RPMI-1640 medium, the human monocyte cell line THP-1 was cultured. First, THP-1 cells were stimulated for 6 hours with 320 nM PWA (Sigma, MO, USA) to differentiate into M0 macrophages. Lipopolysaccharide (LPS; Beyotime, China; 100 ng/mL) was used to stimulate M0 macrophages for 48 hours in order to polarize them into M1 macrophages.

### siRNA transfection

The siRNA was purchased from OBiO Technology (Shanghai). For siRNA transfection, the siRNA sequence used to knock down IL18RAP (si-IL18RAP) was si-IL18RAP-1 (forward sequence: 5’-AAAAUAAGACAAAUUCCUCUU-3’ and reverse sequence: 5’- GAGGAAUUUGUCUUAUUUUGU-3’) and si-IL18RAP-2 (forward sequence: 5’-AUAGCUUUUCCUAAUGUCCUC-3’ and reverse sequence: 5’- GGACAUUAGGAAAAGCUAUCC-3’). According to the manufacturer’s protocol, the U251, MDA-MB-231 and HepG2 were transfected with si-NC, si-IL18RAP-1 or si-IL18RAP-2 using Lipofectamine 2000 (Thermo Fisher, China).

### Immunofluorescence staining

At Renmin Hospital of Wuhan University, tissue samples from hospitalized patients were collected and the pathology department provided all sample wax blocks.

The produced tissue sections were given a 3% H2O2 treatment for 10 minutes and then incubated with the primary antibody at 4° C after being blocked in 3% bovine serum albumin and 0.3% Triton X-100. Following that, a corresponding fluorescent secondary antibody was incubated with the samples. DAPI was used to counterstain nuclei. We processed multiplex immunofluorescence staining using TSA fluorescent kits (Servicebio, China) according the manufacturer’s instructions. Finally, we acquired images using a fluorescence microscope (Olympus BX51). Anti-IL18RAP (1:30; abs111754, Absin), anti-CD68 (1:100; ab213363, Abcam), and anti-iNOS (1:500; ab178945, Abcam) were the primary antibodies employed.

### Western blot analysis

In a word, we obtained the cell samples and lysed them in RIPA buffer (Beyotime, China) with 0.1 mM PMSF (Beyotime, China). After centrifuging the lysate, the supernatant was gathered. The proteins were then transferred to PVDF membranes through electrophoresis. The membranes and the primary antibodies were then incubated together for an overnight period at 4° C after being blocked with 5% nonfat milk and being washed with TBS-T. The membranes were then exposed for an hour to the secondary antibodies. In the end, we used the ChemiDocTM Touch Imaging System to take the images (Bio-Rad, CA, USA). The outcomes were examined with the use of ImageJ software.

### Coculture assay for the migration of M1 macrophages

We added si-NC and si-IL18RAP groups of MDA-MB-231, U251 and HepG2 (5 × 10^5^) to the lower chamber and M1 macrophages (5 × 10^5^) to the upper chamber to conduct cell migration tests. Nonmigrated cells were removed after coculturing for 24 hours, and migrated M1 macrophages were then stained with 0.5% crystal violet solution. Finally, we used an inverted microscope to obtain the images (Olympus). Five areas were randomly selected to count the cells and calculate the mean value. The outcomes were examined with the use of ImageJ software.

### Colony formation assay

MDA-MB-231 cells transfected with si-IL18RAP or si-NC as described above were seeded in 6-well plates at a density of 8×10^2^ cells/well. The MDA-MB-231 cells were cultured for 2 weeks until single-cell colonies formed. MDA-MB-231 cells were fixed with 4% paraformaldehyde for 15 min after washing with phosphate buffer and stained with 0.5% crystal violet solution for 15 min. ImageJ software was used to evaluate the results.

### Transwell migration and invasion assay

MDA-MB-231 cells transfected with siRNA were digested and resuspended; 200 μl of serum-free medium containing 2× 10^3^ cells was added to the upper chamber, and 600 μl of RPMI-1640 medium containing 10% foetal bovine serum (Hangzhou Sijiqing, China) was added to the lower chamber. Nonmigrated cells were removed after coculturing for 24 hours, and migrated MDA-MB-231 cells were then stained with 0.5% crystal violet solution for 15 min. An inverted microscope was used to obtain the images. Five areas were randomly selected to count the cells and calculate the mean value. For invasion assays, Matrigel and serum-free medium were mixed at a ratio of 1:8 and 80μl mixed solution was then added to the upper chamber. The following steps of the experiment were identical to those in the migration experiment. The outcomes were evaluated using ImageJ software.

### Wound-healing assay

MDA-MB-231 cells were plated in 6-well plates until cell confluence reached 80% after si-IL18RAP or si-NC transfection. Cells were scraped with a sterile pipette tip to form a straight line. The culture was then continued with serum-free RPMI-1640 media after the floating cells had been removed with PBS. At 0, 24, and 48 hours, pictures were taken with an inverted microscope. Finally, ImageJ software was used to measure and calculate the percentage of wound healing area at different time points.

### Statistical analysis

For statistical analysis, we utilized R software (version 4.2.1), and to determine the connection between different variables, we used the Spearman method. Using the SPSS 19.0 program, a Student’s t test or a one-way ANOVA was employed to compare the differences (SPSS Inc., Illinois). The graphs were produced using GraphPad Prism 5.0 software. All information is displayed as means ± standard deviations (SD). P < 0.05 indicates a significant difference.

### Data availability statement

The datasets generated for this study are available on request to the corresponding authors.

## Supplementary Material

Supplementary Figures

## References

[1] Siegel RL, Miller KD, Fuchs HE, Jemal A. Cancer Statistics, 2021. CA Cancer J Clin. 2021; 71:7–33. 10.3322/caac.21654 33433946

[2] Sung H, Ferlay J, Siegel RL, Laversanne M, Soerjomataram I, Jemal A, Bray F. Global Cancer Statistics 2020: GLOBOCAN Estimates of Incidence and Mortality Worldwide for 36 Cancers in 185 Countries. CA Cancer J Clin. 2021; 71:209–49. 10.3322/caac.21660 33538338

[3] Bagchi S, Yuan R, Engleman EG. Immune Checkpoint Inhibitors for the Treatment of Cancer: Clinical Impact and Mechanisms of Response and Resistance. Annu Rev Pathol. 2021; 16:223–49. 10.1146/annurev-pathol-042020-042741 33197221

[4] Carlino MS, Larkin J, Long GV. Immune checkpoint inhibitors in melanoma. Lancet. 2021; 398:1002–14. 10.1016/S0140-6736(21)01206-X 34509219

[5] Hussaini S, Chehade R, Boldt RG, Raphael J, Blanchette P, Maleki Vareki S, Fernandes R. Association between immune-related side effects and efficacy and benefit of immune checkpoint inhibitors - A systematic review and meta-analysis. Cancer Treat Rev. 2021; 92:102134. 10.1016/j.ctrv.2020.102134 33302134

[6] Dinarello CA, Novick D, Puren AJ, Fantuzzi G, Shapiro L, Mühl H, Yoon DY, Reznikov LL, Kim SH, Rubinstein M. Overview of interleukin-18: more than an interferon-gamma inducing factor. J Leukoc Biol. 1998; 63:658–64. 9620656

[7] Okamura H, Tsutsi H, Komatsu T, Yutsudo M, Hakura A, Tanimoto T, Torigoe K, Okura T, Nukada Y, Hattori K. Cloning of a new cytokine that induces IFN-gamma production by T cells. Nature. 1995; 378:88–91. 10.1038/378088a0 7477296

[8] Okamura H, Kashiwamura S, Tsutsui H, Yoshimoto T, Nakanishi K. Regulation of interferon-gamma production by IL-12 and IL-18. Curr Opin Immunol. 1998; 10:259–64. 10.1016/s0952-7915(98)80163-5 9638361

[9] Takeda K, Tsutsui H, Yoshimoto T, Adachi O, Yoshida N, Kishimoto T, Okamura H, Nakanishi K, Akira S. Defective NK cell activity and Th1 response in IL-18-deficient mice. Immunity. 1998; 8:383–90. 10.1016/s1074-7613(00)80543-9 9529155

[10] Dinarello CA. IL-18: A TH1-inducing, proinflammatory cytokine and new member of the IL-1 family. J Allergy Clin Immunol. 1999; 103:11–24. 10.1016/s0091-6749(99)70518-x 9893178

[11] Hoeve MA, Savage ND, de Boer T, Langenberg DM, de Waal Malefyt R, Ottenhoff TH, Verreck FA. Divergent effects of IL-12 and IL-23 on the production of IL-17 by human T cells. Eur J Immunol. 2006; 36:661–70. 10.1002/eji.200535239 16482511

[12] Leite-De-Moraes MC, Hameg A, Pacilio M, Koezuka Y, Taniguchi M, Van Kaer L, Schneider E, Dy M, Herbelin A. IL-18 enhances IL-4 production by ligand-activated NKT lymphocytes: a pro-Th2 effect of IL-18 exerted through NKT cells. J Immunol. 2001; 166:945–51. 10.4049/jimmunol.166.2.945 11145671

[13] Born TL, Thomassen E, Bird TA, Sims JE. Cloning of a novel receptor subunit, AcPL, required for interleukin-18 signaling. J Biol Chem. 1998; 273:29445–50. 10.1074/jbc.273.45.29445 9792649

[14] Cheung H, Chen NJ, Cao Z, Ono N, Ohashi PS, Yeh WC. Accessory protein-like is essential for IL-18-mediated signaling. J Immunol. 2005; 174:5351–7. 10.4049/jimmunol.174.9.5351 15843532

[15] Reijmerink NE, Postma DS, Bruinenberg M, Nolte IM, Meyers DA, Bleecker ER, Koppelman GH. Association of IL1RL1, IL18R1, and IL18RAP gene cluster polymorphisms with asthma and atopy. J Allergy Clin Immunol. 2008; 122:651–4.e8. 10.1016/j.jaci.2008.06.030 18774397

[16] Cherlin S, Lewis MJ, Plant D, Nair N, Goldmann K, Tzanis E, Barnes MR, McKeigue P, Barrett JH, Pitzalis C, Barton A, Cordell HJ, and MATURA Consortium. Investigation of genetically regulated gene expression and response to treatment in rheumatoid arthritis highlights an association between *IL18RAP* expression and treatment response. Ann Rheum Dis. 2020; 79:1446–52. 10.1136/annrheumdis-2020-217204 32732242 PMC7569378

[17] Festen EA, Goyette P, Green T, Boucher G, Beauchamp C, Trynka G, Dubois PC, Lagacé C, Stokkers PC, Hommes DW, Barisani D, Palmieri O, Annese V, et al. A meta-analysis of genome-wide association scans identifies IL18RAP, PTPN2, TAGAP, and PUS10 as shared risk loci for Crohn’s disease and celiac disease. PLoS Genet. 2011; 7:e1001283. 10.1371/journal.pgen.1001283 21298027 PMC3029251

[18] Zhu J, Liu C, Teng X, Yin J, Zheng L, Wang L, Tang W, Gu H, Gu B, Chen L. Association of the interleukin-18 receptor 1 and interleukin-18 receptor accessory protein polymorphisms with the risk of esophageal cancer. Biomed Rep. 2016; 4:227–35. 10.3892/br.2015.552 26893844 PMC4734065

[19] Wang T, Chen B, Meng T, Liu Z, Wu W. Identification and immunoprofiling of key prognostic genes in the tumor microenvironment of hepatocellular carcinoma. Bioengineered. 2021; 12:1555–75. 10.1080/21655979.2021.1918538 33955820 PMC8806269

[20] Li Z, Yu X, Werner J, Bazhin AV, D’Haese JG. The role of interleukin-18 in pancreatitis and pancreatic cancer. Cytokine Growth Factor Rev. 2019; 50:1–12. 10.1016/j.cytogfr.2019.11.001 31753718

[21] Nakamura K, Kassem S, Cleynen A, Chrétien ML, Guillerey C, Putz EM, Bald T, Förster I, Vuckovic S, Hill GR, Masters SL, Chesi M, Bergsagel PL, et al. Dysregulated IL-18 Is a Key Driver of Immunosuppression and a Possible Therapeutic Target in the Multiple Myeloma Microenvironment. Cancer Cell. 2018; 33:634–48.e5. 10.1016/j.ccell.2018.02.007 29551594

[22] Markowitz GJ, Yang P, Fu J, Michelotti GA, Chen R, Sui J, Yang B, Qin WH, Zhang Z, Wang FS, Diehl AM, Li QJ, Wang H, Wang XF. Inflammation-Dependent IL18 Signaling Restricts Hepatocellular Carcinoma Growth by Enhancing the Accumulation and Activity of Tumor-Infiltrating Lymphocytes. Cancer Res. 2016; 76:2394–405. 10.1158/0008-5472.CAN-15-1548 26893476 PMC4873403

[23] Zhuang W, Sun H, Zhang S, Zhou Y, Weng W, Wu B, Ye T, Huang W, Lin Z, Shi L, Shi K. An immunogenomic signature for molecular classification in hepatocellular carcinoma. Mol Ther Nucleic Acids. 2021; 25:105–15. 10.1016/j.omtn.2021.06.024 34401208 PMC8332372

[24] Takemoto A, Tanimoto K, Mori S, Inoue J, Fujiwara N, Noda T, Inazawa J. Integrative genome-wide analyses reveal the transcriptional aberrations in Japanese esophageal squamous cell carcinoma. Cancer Sci. 2021; 112:4377–92. 10.1111/cas.15063 34263978 PMC8486213

[25] Zarrei M, MacDonald JR, Merico D, Scherer SW. A copy number variation map of the human genome. Nat Rev Genet. 2015; 16:172–83. 10.1038/nrg3871 25645873

[26] Hinshaw DC, Shevde LA. The Tumor Microenvironment Innately Modulates Cancer Progression. Cancer Res. 2019; 79:4557–66. 10.1158/0008-5472.CAN-18-3962 31350295 PMC6744958

[27] Fridman WH, Zitvogel L, Sautès-Fridman C, Kroemer G. The immune contexture in cancer prognosis and treatment. Nat Rev Clin Oncol. 2017; 14:717–34. 10.1038/nrclinonc.2017.101 28741618

[28] Myers JA, Miller JS. Exploring the NK cell platform for cancer immunotherapy. Nat Rev Clin Oncol. 2021; 18:85–100. 10.1038/s41571-020-0426-7 32934330 PMC8316981

[29] McLane LM, Abdel-Hakeem MS, Wherry EJ. CD8 T Cell Exhaustion During Chronic Viral Infection and Cancer. Annu Rev Immunol. 2019; 37:457–95. 10.1146/annurev-immunol-041015-055318 30676822

[30] Vidal-Vanaclocha F, Mendoza L, Telleria N, Salado C, Valcárcel M, Gallot N, Carrascal T, Egilegor E, Beaskoetxea J, Dinarello CA. Clinical and experimental approaches to the pathophysiology of interleukin-18 in cancer progression. Cancer Metastasis Rev. 2006; 25:417–34. 10.1007/s10555-006-9013-3 17001512

[31] Cassetta L, Pollard JW. Targeting macrophages: therapeutic approaches in cancer. Nat Rev Drug Discov. 2018; 17:887–904. 10.1038/nrd.2018.169 30361552

[32] Vitale I, Manic G, Coussens LM, Kroemer G, Galluzzi L. Macrophages and Metabolism in the Tumor Microenvironment. Cell Metab. 2019; 30:36–50. 10.1016/j.cmet.2019.06.001 31269428

[33] Kinker GS, Greenwald AC, Tal R, Orlova Z, Cuoco MS, McFarland JM, Warren A, Rodman C, Roth JA, Bender SA, Kumar B, Rocco JW, Fernandes PA, et al. Pan-cancer single-cell RNA-seq identifies recurring programs of cellular heterogeneity. Nat Genet. 2020; 52:1208–18. 10.1038/s41588-020-00726-6 33128048 PMC8135089

[34] Qian J, Olbrecht S, Boeckx B, Vos H, Laoui D, Etlioglu E, Wauters E, Pomella V, Verbandt S, Busschaert P, Bassez A, Franken A, Bempt MV, et al. A pan-cancer blueprint of the heterogeneous tumor microenvironment revealed by single-cell profiling. Cell Res. 2020; 30:745–62. 10.1038/s41422-020-0355-0 32561858 PMC7608385

[35] Sareneva T, Julkunen I, Matikainen S. IFN-alpha and IL-12 induce IL-18 receptor gene expression in human NK and T cells. J Immunol. 2000; 165:1933–8. 10.4049/jimmunol.165.4.1933 10925275

[36] Salvati VM, MacDonald TT, Bajaj-Elliott M, Borrelli M, Staiano A, Auricchio S, Troncone R, Monteleone G. Interleukin 18 and associated markers of T helper cell type 1 activity in coeliac disease. Gut. 2002; 50:186–90. 10.1136/gut.50.2.186 11788557 PMC1773110

[37] Dolina JS, Van Braeckel-Budimir N, Thomas GD, Salek-Ardakani S. CD8^+^ T Cell Exhaustion in Cancer. Front Immunol. 2021; 12:715234. 10.3389/fimmu.2021.715234 34354714 PMC8330547

[38] Cha JH, Chan LC, Li CW, Hsu JL, Hung MC. Mechanisms Controlling PD-L1 Expression in Cancer. Mol Cell. 2019; 76:359–70. 10.1016/j.molcel.2019.09.030 31668929 PMC6981282

[39] Budimir N, Thomas GD, Dolina JS, Salek-Ardakani S. Reversing T-cell Exhaustion in Cancer: Lessons Learned from PD-1/PD-L1 Immune Checkpoint Blockade. Cancer Immunol Res. 2022; 10:146–53. 10.1158/2326-6066.CIR-21-0515 34937730

[40] van der Leun AM, Thommen DS, Schumacher TN. CD8^+^ T cell states in human cancer: insights from single-cell analysis. Nat Rev Cancer. 2020; 20:218–32. 10.1038/s41568-019-0235-4 32024970 PMC7115982

