# Supplementary material for: Prognostic and immunological roles of IL18RAP in human cancers
Source: Aging (Albany NY). 2023 Sep 11;15(17):9059–85. doi: 10.18632/aging.205017 (PMC10522399; doi:10.18632/aging.205017)
Supplement: Supplementary Figures [file aging-15-205017-s001.pdf]

SUPPLEMENTARY FIGURES

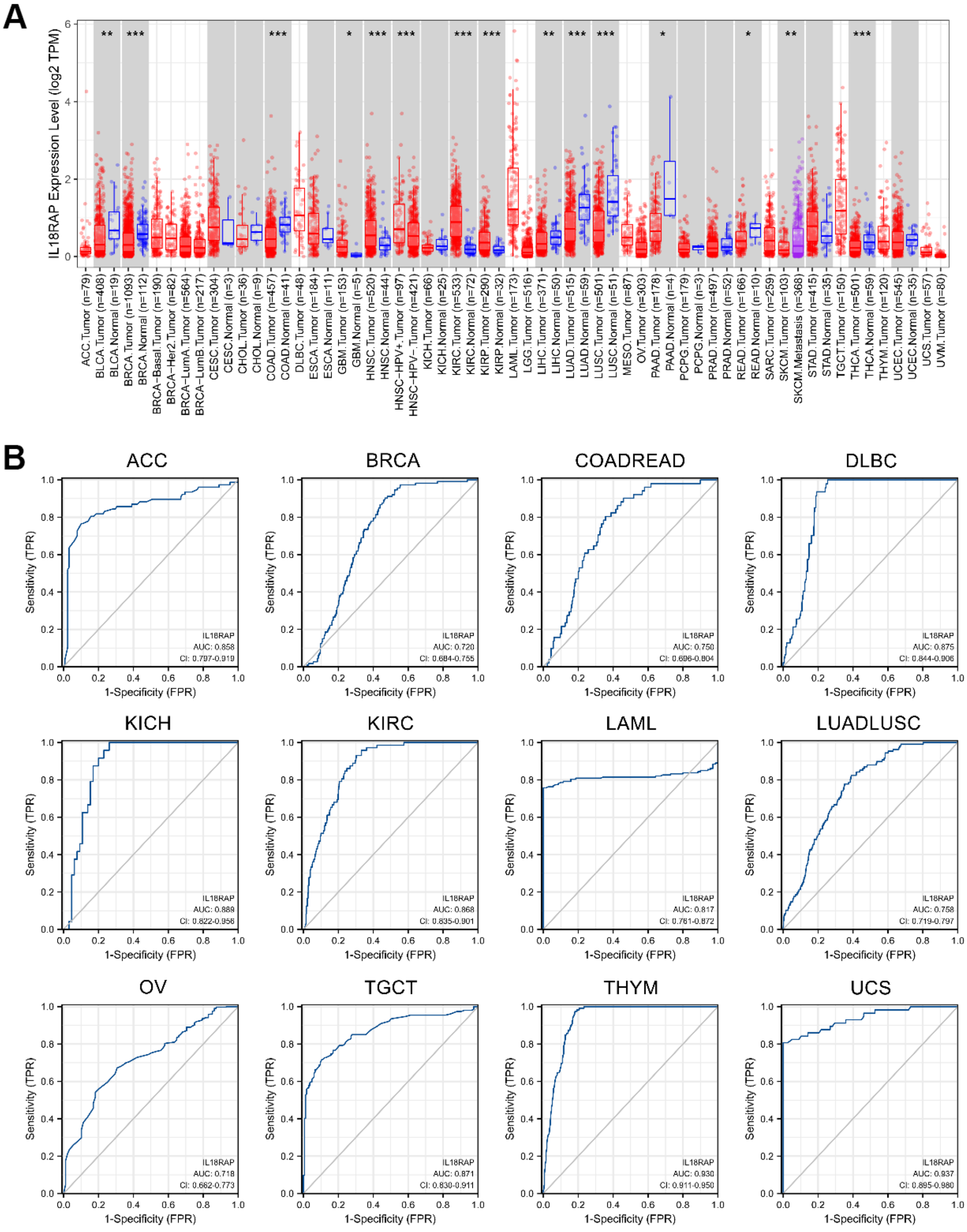

Supplementary Figure 1. The differential expression and diagnostic analysis of IL18RAP in pan-cancer. (**A**) The expression of IL18RAP in pan-cancer obtained from TIMER2.0 database. *p < 0.05; **p < 0.01 and ***p < 0.001. (**B**) Receiver Operator Characteristic (ROC) curve of IL18RAP in ACC, BRCA, COADREAD, DLBC, KICH, KIRC, LAML, LUADLUSC, OV, TGCT, THYM, and UCS. The AUC value (0.5- 0.7, 0.7 - 0.9, 0.9-1) represents low, good and high accuracy respectively.

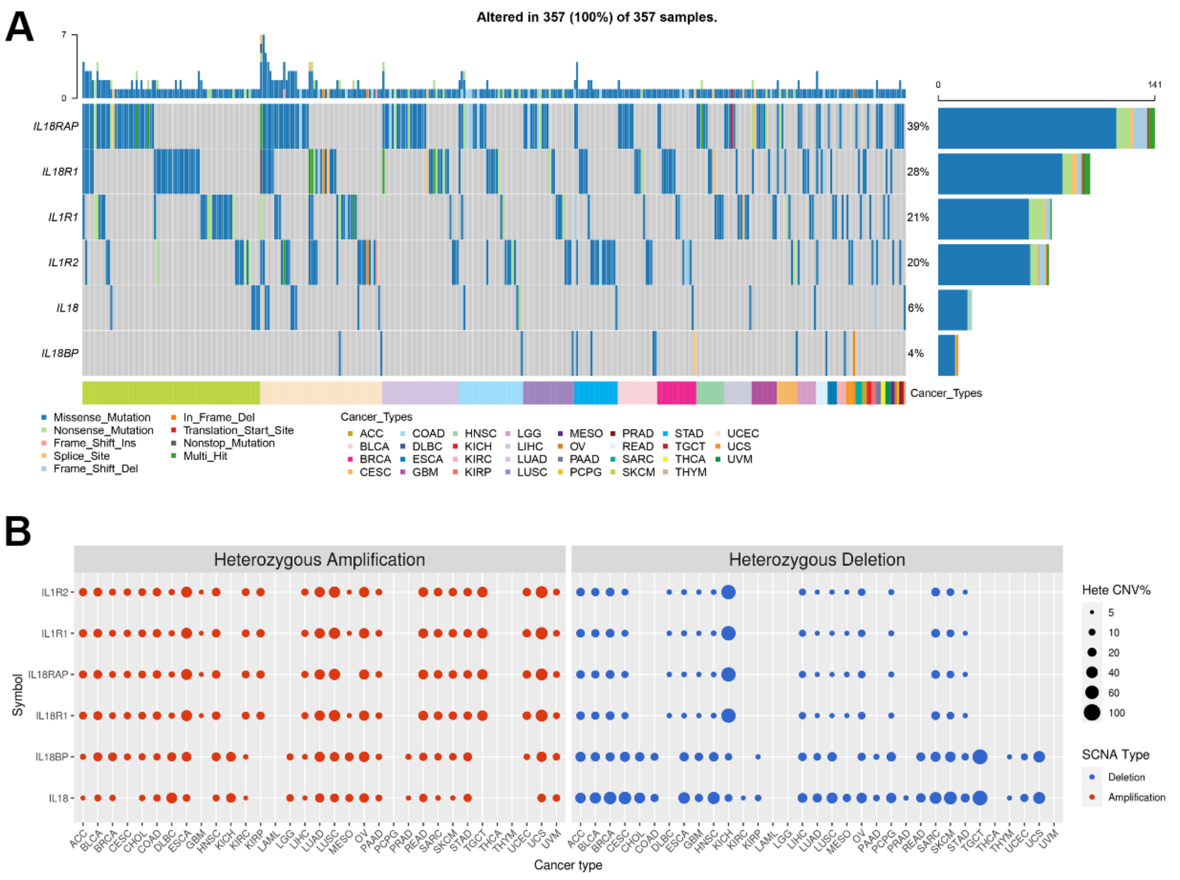

Supplementary Figure 2. **Genetic alterations of IL1R1, IL1R2, IL18, IL18R1, IL18BP, and IL18RAP in pan-cancer.** (**A**) The mutation frequency and types of IL1R1, IL1R2, IL18, IL18R1, IL18BP, and IL18RAP in 31 cancers. (**B**) The heterozygous amplification (left) and deletion (right) of IL18RAP and other 5 genes in pan-cancer. The analyses were based on GSCALite platform.

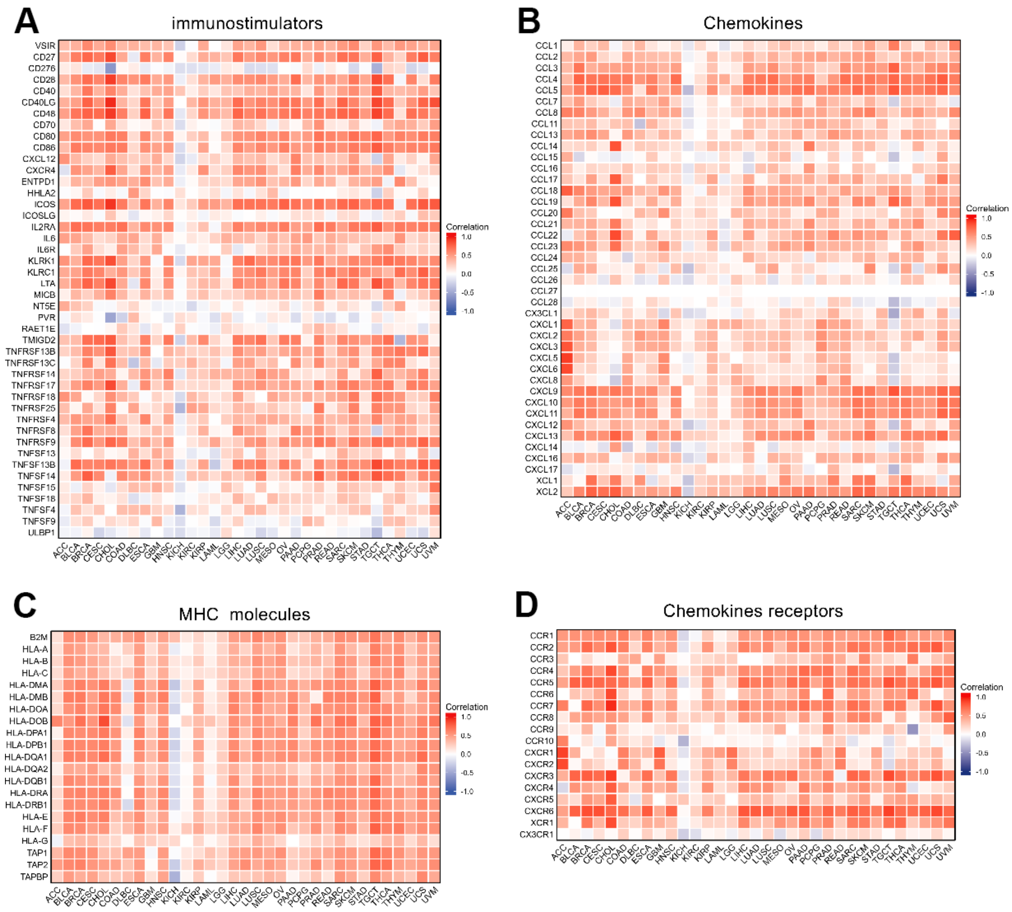

Supplementary Figure 3. Correlation of IL18RAP with immunostimulators, MHC molecules, chemokines and chemokine receptors genes in 33 cancers. (**A**–**D**) The correlation analysis between IL18RAP expression and Immunostimulators (**A**), Chemokines (**B**), MHC molecules (**C**), Chemokine receptors (**D**) genes using Spearman correlation analysis.

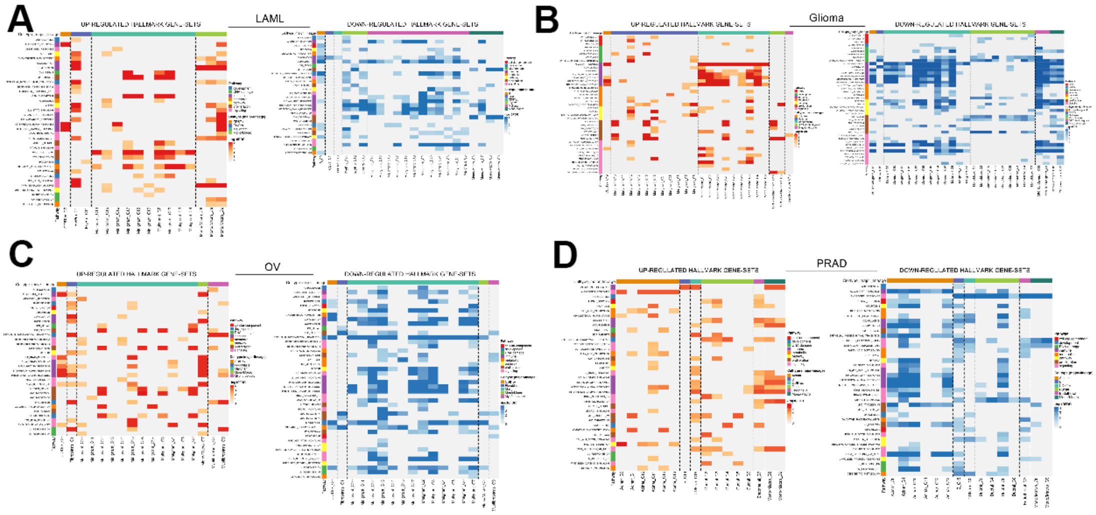

Supplementary Figure 4. The HALLMARK gene sets analysis on each cell clusters based on 4 datasets. (**A**–**D**) The HALLMARK gene sets analysis on each cell clusters based on LAML (GSE154109) (**A**), Glioma (GSE131928_10X) (**B**), OV (GSE130000) (**C**), and PRAD (GSE150692) (**D**) datasets. The analyses were based on TISCH2 platform.

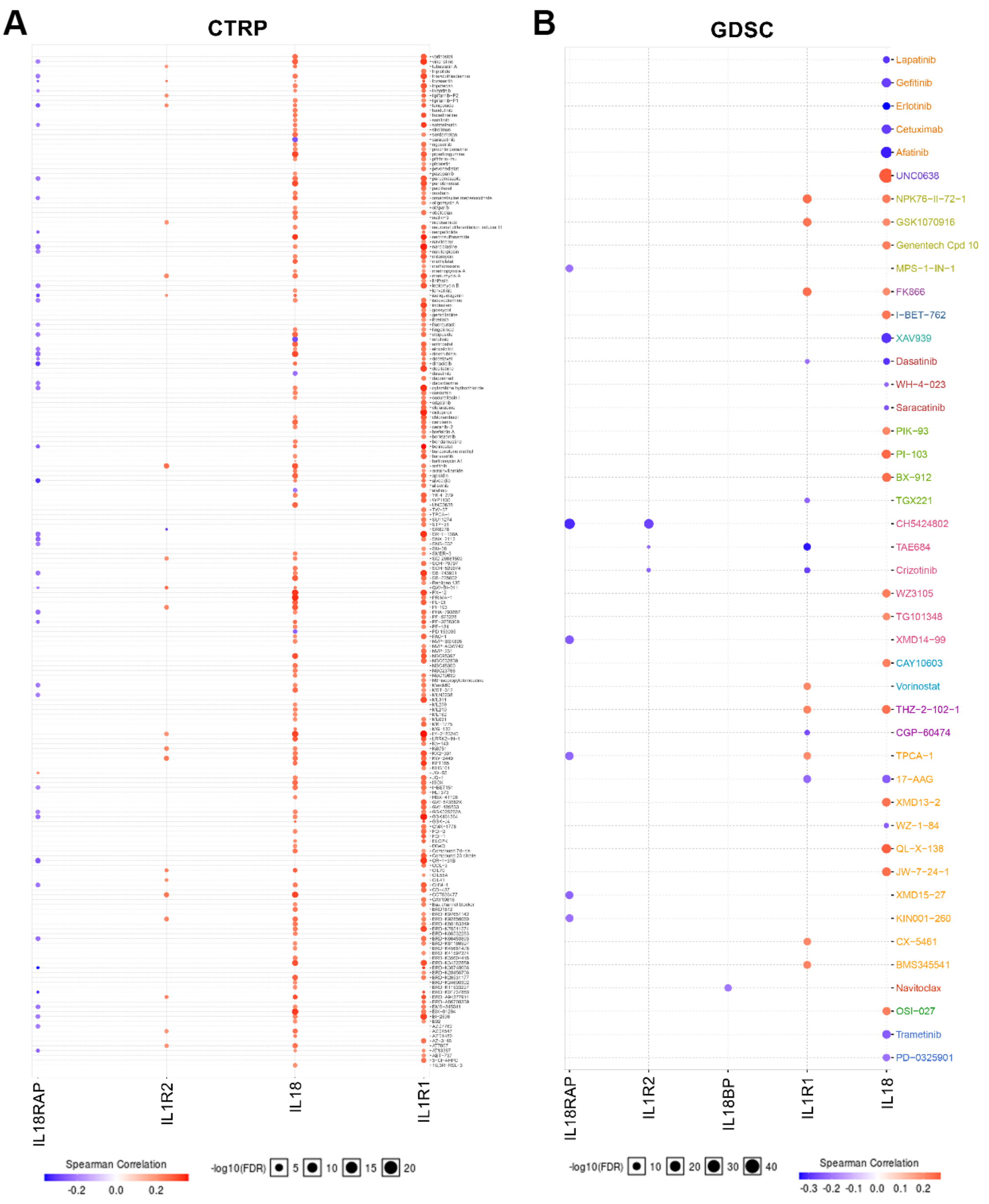

Supplementary Figure 5. The drug sensitivity of IL18RAP in cancers. (**A**, **B**) The correlation analysis between IL18RAP expression and IC50 level of various drugs in cancer cell lines based on the CTRP (**A**) and GDSC (**B**) databases. The analyses were based on GSCALite platform.
